# *CAGEr*: precise TSS data retrieval and high-resolution promoterome mining for integrative analyses

**DOI:** 10.1093/nar/gkv054

**Published:** 2015-02-04

**Authors:** Vanja Haberle, Alistair R.R. Forrest, Yoshihide Hayashizaki, Piero Carninci, Boris Lenhard

**Affiliations:** 1 Department of Biology, University of Bergen, Thormøhlensgate 53 A & B, N–5008 Bergen, Norway; 2 Department of Molecular Sciences, Institute of Clinical Sciences, Faculty of Medicine, Imperial College London and MRC Clinical Sciences Centre, Hammersmith Hospital Campus, Du Cane Road, London W12 0NN, UK; 3 RIKEN Center for Life Science Technologies, Division of Genomic Technologies (CLST DGT), RIKEN Yokohama Campus, 1-7-22 Suehiro-cho, Tsurumi-ku, Yokohama, Kanagawa 230-0045, Japan; 4 RIKEN Preventive Medicine and Diagnosis Innovation Program (PMI), 2-1 Hirosawa, Wako-shi, Saitama 351-0198, Japan; 5 Department of Informatics, University of Bergen, Høyteknologisenteret, Thormøhlensgate 55, N–5008 Bergen, Norway

## Abstract

Cap analysis of gene expression (CAGE) is a high-throughput method for transcriptome analysis that provides a single base-pair resolution map of transcription start sites (TSS) and their relative usage. Despite their high resolution and functional significance, published CAGE data are still underused in promoter analysis due to the absence of tools that enable its efficient manipulation and integration with other genome data types. Here we present *CAGEr*, an R implementation of novel methods for the analysis of differential TSS usage and promoter dynamics, integrated with CAGE data processing and promoterome mining into a first comprehensive CAGE toolbox on a common analysis platform. Crucially, we provide collections of TSSs derived from most published CAGE datasets, as well as direct access to FANTOM5 resource of TSSs for numerous human and mouse cell/tissue types from within R, greatly increasing the accessibility of precise context-specific TSS data for integrative analyses. The *CAGEr* package is freely available from Bioconductor at http://www.bioconductor.org/packages/release/bioc/html/CAGEr.html.

## INTRODUCTION

The transcription of protein-coding RNA (mRNA) and several classes of non-coding RNAs is initiated by RNA Polymerase II (RNAPII) complex at discrete loci known as RNAPII promoters (1). They are the sites of binding and positioning of the machinery that initiates transcription at individual nucleotides called transcription start sites (TSS). Mapping of 5′ ends of individual mRNAs by oligo-capping and genome-wide by cap analysis of gene expression (CAGE), revealed that the transcription can start at multiple closely spaced TSSs within a promoter (2,3) challenging the traditional view of a gene promoter and its precisely defined TSS.

**Figure 1.**
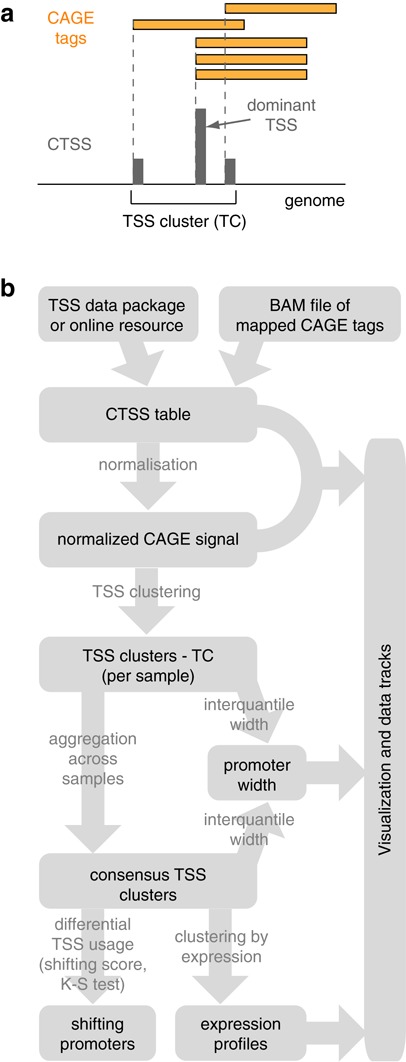
*CAGEr* workflow. (**a**) Schematic representation of CAGE data and explanation of key terms. (**b**) Flow chart of main steps in *CAGEr*. CTSS, CAGE detected TSS; TC, tag/TSS cluster.

CAGE is a high-throughput method for transcriptome analysis that captures the 5′ end of the transcribed and capped mRNAs (4). Sequencing of short fragments from the very 5′ end yields a large number of CAGE tags that can be mapped back to the reference genome to infer the exact position of the TSSs of captured RNAs. The number of CAGE tags supporting each TSS reflects the relative frequency of its usage and can be used as a measure of expression from that specific TSS (5). Thus, CAGE provides information on two aspects of the capped transcriptome: (i) genome-wide single base-pair resolution map of TSSs and (ii) relative levels of transcripts initiated at each TSS (Figure 1a). This information can be used for various analyses, from studying promoter architecture (2,6) to 5′ end-centred expression profiling (7,8).

Mapping genome-wide TSSs by CAGE in a vast number of mouse and human cell and tissue types (9–11) led to the discovery of distinct classes of promoters with respect to TSS distribution. They differ in underlying sequence features and associated gene function (2), and are subject to distinct modes of regulation (reviewed in (12)). CAGE has also been used to identify key transcription factors binding at promoters, and to reconstruct the regulatory networks that drive the differentiation (8) and maintain cellular identity (11), as well as to construct an atlas of active enhancers across the whole human body (13). Thus, in addition to providing a valuable resource of genome-wide cell type-specific TSSs, as a more precise and context-sensitive alternative to TSS positions available in annotation databases, CAGE is also a powerful approach for studying various aspects of gene regulation.

The initial studies of genome-wide CAGE datasets have introduced basic methods for processing sequenced and mapped CAGE tags dealing with removal of protocol specific G nucleotide addition bias (2) and precise TSS calling. Different clustering approaches have been used to reconstruct promoters, based either on the fixed distance between individual TSSs (2,7) or on the density of transcription initiation events (14). With the increase of sequencing depth, normalization approaches and noise modelling have also been introduced (15) to enable expression profiling from CAGE. On the other hand, the high-resolution positional information has been used to analyse the distribution of TSSs within promoters, with various measures devised to assess promoter width and shape (2,6,14). Furthermore, the first genome-wide investigation of differential TSS usage within individual promoters detected extensive positional and/or regional bias in TSS usage across multiple tissues (16) emphasizing the importance of context-specific TSS information. Despite various methods used for analysing CAGE data, and several recently published programs that address specific questions in CAGE data analysis (17,18), no software package has been published that would integrate a comprehensive CAGE workflow with an easy access to a growing resource of CAGE-detected TSSs on a commonly used analysis platform, allowing users to integrate high-resolution TSS data with other genome-wide data types. For that reason, the available CAGE data has been under-utilized relative to its power, resolution and the amount of precision it brings into the analysis of promoter structure and function, in favour of less precise annotation.

Here we present *CAGEr*, a freely available R/Bioconductor package that implements various methods for CAGE data processing and promoterome mining and provides access to majority of published CAGE datasets in several organisms (6,9,10,19,20), including the recent FANTOM5 collection of TSSs for numerous human and mouse cell and tissue types (11). *CAGEr* further introduces methods for the analysis of differential TSS usage and detection of ‘shifting’ promoters, a novel concept addressing variability in the choice of TSSs within the same promoter between different contexts (21). To demonstrate the provided functionality and various outputs produced by *CAGEr*, we apply the workflow to a previously unanalysed set of eight CAGE datasets covering mouse testis development from embryonic day 13 to adulthood produced by the FANTOM5 Consortium (11), and we reveal extensive differential TSS usage within individual promoter region between early embryonic and adult testis.

## MATERIALS AND METHODS

### The *CAGEr* package

*CAGEr* is a software package developed for the R computing and statistical environment (22) and is distributed within the Bioconductor project (23) at http://www.bioconductor.org/packages/release/bioc/html/CAGEr.html. The source code of the package is also available from http://promshift.genereg.net/CAGEr/PackageSource/. The package provides functionality for processing and analysing CAGE data starting from different input formats, through a workflow consisting of successive, well-documented steps. Detailed description of each function and comprehensive user guide with example analysis are distributed with the package and are also provided here in Supplementary Methods. *CAGEr* starts from sequenced and mapped CAGE tags and performs quality filtering and removal of protocol-specific 5′ end G nucleotide addition bias to identify precise TSS positions and frequency of their usage. Alternatively, already called single base-pair resolution TSSs, provided by the user or retrieved from one of the available resources described below, can be used as input and included into the workflow. Several normalization methods of raw CAGE tag counts are supported and accompanied by graphical outputs that aid in selecting optimal parameters for normalization. *CAGEr* further constructs context-specific promoterome by clustering individual TSSs into tag clusters (TC) using one of the several supported clustering approaches. It manipulates multiple CAGE experiments at once, performs expression profiling across experiments, both at the level of individual TSSs and clusters of TSSs, and exports several different types of track files for visualization in the genome browser. Implementation of assessment of promoter width is provided, which uses interquantile width as a measure of width robust to expression level, which allows classification of promoters into sharp or broad class. *CAGEr* also introduces novel method for detection of differential TSS usage, addressing the variability in TSS choice and promoter shifting between different contexts. The context-specific promoterome with precise TSS positions and various additional layers of information constructed using *CAGEr* can be integrated into any promoter-centred analysis. To facilitate the reuse of available public CAGE data, *CAGEr* provides access to TSSs for numerous human and mouse samples from FANTOM5 collection, which are retrieved from the FANTOM5 online resource (http://fantom.gsc.riken.jp/5/datafiles/latest/basic/) and are imported directly into the workflow in R. The list and short description of all human and mouse FANTOM5 samples is available in *CAGEr* and can be used to search and retrieve TSS data for selected samples (example code in Supplementary Methods).

### Mouse testis data

To demonstrate the functionality of the package we used a previously uncharacterized time-course of eight mouse testis CAGE samples produced by the FANTOM5 consortium (11). These include testis samples from embryonic days 13, 15 and 17, neonate days 0, 10, 20 and 30, and from an adult mouse. Tab-separated flat files with genomic positions of CAGE-detected TSSs and associated tag counts mapped to the mm9 assembly of the mouse genome were obtained from the FANTOM5 web resource (http://fantom.gsc.riken.jp/5/datafiles/latest/basic/mouse.tissue.hCAGE/) and were used as input for *CAGEr*. The TSS input data is available from http://promshift.genereg.net/CAGEr/InputData/ and documented R code for processing these data with *CAGEr* and performing analyses presented in this paper is provided in Supplementary Methods.

BioCap data for non-methylated regions in mouse testis produced by Long *et al*. (24) were obtained from GEO (accession code: GSM1064678) and coordinates of CpG islands for mm9 genome assembly were downloaded from the UCSC Genome Browser. Position weight matrix for TATA-box motif was downloaded from the Jaspar database (25) and used to score the region from −35 to −22 bp upstream of the dominant TSS in sharp and broad promoters. RefSeq gene annotation for mm9 genome assembly was obtained from the UCSC Genome Browser and was associated with the closest CAGE-derived TSS cluster falling within −1000 to +500 bp from the annotated TSS.

### R data packages containing FANTOM, ENCODE and zebrafish CAGE data

We have collected publicly available CAGE datasets produced by the FANTOM consortium in the FANTOM3 and FANTOM4 projects (8–10) and organized the detected TSSs into *FANTOM3and4CAGE* R data package. The package contains data for various human and mouse tissues and several time-courses. Each dataset within the package provides genomic coordinates of TSSs detected by CAGE in a group of related samples, along with the number of supporting CAGE tags in each individual sample. This package is freely available through Bioconductor (23) at http://www.bioconductor.org/packages/release/data/experiment/html/FANTOM3and4CAGE.html.

We provide an analogous R data package containing TSSs derived from ENCODE CAGE data (19) for various human cell lines. The format of CAGE data provided by ENCODE at UCSC includes only raw mapped CAGE tags, their coverage along the genome and the coordinates of the enriched genomic regions (peaks), which do not take advantage of the single base-pair resolution TSS information provided by CAGE. To address this, we have processed mapped CAGE tags with *CAGEr*, removed the 5′ end G nucleotide addition bias and derived single base-pair resolution TSSs, which were then collected into an R data package named *ENCODEprojectCAGE*. The package also includes modENCODE project CAGE dataset for fruit fly (*Drosophila melanogaster*) embryos (6). This data package is freely available for download from http://promshift.genereg.net/CAGEr/PackageSource/ and is accompanied by a user manual explaining its content and usage.

Our previously published CAGE data for 12 developmental stages of zebrafish (20) (*Danio rerio*) has also been collected into a data package that can be used with *CAGEr*. The *ZebrafishDevelopmentalCAGE* package and accompanying user manual are available for download from http://promshift.genereg.net/CAGEr/PackageSource/.

Once any of the above mentioned R packages has been downloaded and installed, selected samples can be easily imported into *CAGEr* workflow as exemplified by the R code in Supplementary Methods. This allows users to easily obtain context-specific list of promoters with precise TSS positions and additional promoter information that can be used for integrative analyses.

## RESULTS

### CAGEr workflow overview

The workflow provided by *CAGEr* package consists of successive steps of TSS data processing and more complex downstream analyses (Figure 1b), which enable users to obtain comprehensive list of promoters and various associated information by invoking only several well-documented commands (see Supplementary Methods for detailed user guide). Three different formats of input data are supported: (i) binary alignment files of CAGE tags mapped to a reference genome, (ii) table of genomic positions of CAGE-derived TSSs with counts of supporting tags in one or more samples as flat tab-separated file(s) and (iii) direct import of publicly available CAGE datasets from FANTOM5 web resource or from accompanying R data packages (Supplementary Figure S1a). Raw mapped CAGE tags require quality filtering before reliable TSS positions can be derived. In the CAGE experimental protocol an additional G nucleotide is often attached to the 5′ end of the tag by a template-free activity of the reverse transcriptase during cDNA preparation (26), which creates a bias that can be corrected only after mapping. *CAGEr* enables correction of this bias either by using a simple approach of removing the first nucleotide from the tag in case it is a G and does not map to the corresponding genomic sequence, or by applying a systematic probability-based correction algorithm (2). Once the exact 5′ ends of the CAGE tags are established, precise TSSs and supporting tag counts can be called. Individual TSSs and their relative usage can be visualized in the genomic context by exporting the strand-specific single-nucleotide resolution data to a track file format that can be used in any genome browser (Supplementary Figure S1b). A general overview of the datasets and the relationship between different samples can also be obtained by plotting correlation of tag counts per TSS (Supplementary Figure S1c). Various graphical outputs are produced at each step in the workflow, allowing quality checks and driving hypothesis generation. All functionality provided in *CAGEr* is demonstrated here by applying the workflow to eight CAGE samples of mouse testis development (11) (input TSS data available from http://promshift.genereg.net/CAGEr/InputData/ and documented R code provided in Supplementary Methods). A detailed step-by-step user guide through the *CAGEr* workflow with accompanying code snippets is provided in the vignette (Supplementary Methods), which is distributed with the package.

### Tag count normalization

**Figure 2.**
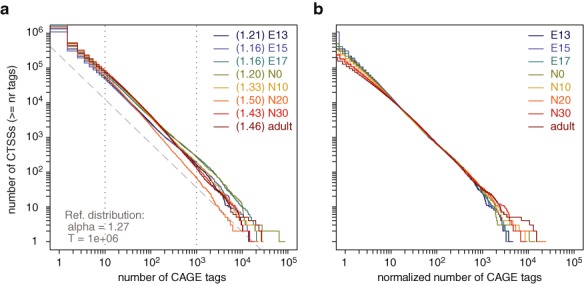
Power-law based normalization (**a**) Reverse cumulative distribution of CAGE tag count per CTSS for eight mouse testis samples plotted with *CAGEr*. Slope of the power-law fitted within the range marked by the dotted lines is shown for each sample in the brackets next to the sample name. Suggested reference power-law distribution is shown as dashed grey line and the corresponding parameters for normalization are denoted in the lower left corner. alpha, absolute value of the reference slope on the log-log scale; T, total number of CAGE tags in the reference distribution. (**b**) Reverse cumulative distribution of CAGE signal per CTSS after normalization. E13 – E17, embryonic day 13–17; N0–N30, neonate day 0–30.

To quantify the expression from each individual TSS and enable comparison between multiple samples, raw tag counts have to be normalized. Many studies performing expression profiling based on CAGE data used number of tags per million (tpm) (7,19,27), which is a simple normalized measure still widely used in many other high-throughput sequencing tag-based studies. However, a systematic investigation of multiple CAGE datasets has revealed that the reverse cumulative distribution of the number of tags per TSS follows a power-law distribution to a very good approximation. Thus, a normalization method that transforms CAGE tag counts in different samples to match a common reference power-law distribution was proposed (15). *CAGEr* supports both normalization methods and provides visualization of reverse cumulative distributions, which aids in deciding on the appropriate normalization approach and in choosing optimal parameters. Figure 2 demonstrates output produced by *CAGEr* showing reverse cumulatives of CAGE signal for eight mouse testis samples (code in Supplementary Methods). The slopes of the power-laws fitted within a specified range of tag count values are reported for each sample and are used to calculate optimal parameters for normalization. The slope of the suggested reference distribution (alpha) is calculated as a median of slopes fitted to individual samples, and the total number of tags (T) is chosen to be the power of 10 closest to the median sequencing depth of the samples (typically 1 million to give normalized tags per million). After normalization, all samples follow the same reference power-law distribution across several orders of magnitude (Figure 2b). Normalized number of CAGE tags can be used to perform expression profiling of individual TSSs to obtain classes of TSSs with the same expression pattern across samples. Finally, the option of performing no normalization at the individual TSS level is also provided, which allows later normalization at the entire promoter level (11) by applying statistical approaches that require raw tag counts (e.g. DEseq (28); edgeR (29)). This enables integration of *CAGEr* workflow with other expression analysis methods available in R.

### TSS clustering and promoterome construction

To reconstruct promoters, individual TSSs are clustered together along the genome. TCs were initially introduced to group together overlapping CAGE tags (9), which resulted in clustering neighbouring TSSs that are less than the length of one tag apart (Figure 1a). This simple distance-based approach was widely used in the following studies and, combined together with the multiple-level clustering, proved to be useful for roughly reconstructing individual gene promoters and analysing their properties (2). However, this approach sets an arbitrary cut-off on the maximal allowed distance between neighbouring TSSs and does not necessarily reflect the intrinsic clustering of the data. This was addressed by introducing a parametric clustering algorithm that attempts to find segments of the genome, which maximize the number of transcription initiation events per nucleotide (14). It finds nested clusters across all possible density values and addresses the hierarchical organization of transcription initiation along the genome (Supplementary Figure S2). In addition to these genome-wide data-driven clustering approaches, *CAGEr* allows TSSs to be distributed into a set of predefined genomic regions, e.g. user-defined windows flanking annotated TSSs. This option enables the refinement of annotation with precise context-specific TSSs. Main characteristics of described clustering approaches are summarized in Table 1. They can all be run in *CAGEr* with a single command that results in a set of clusters per sample with denoted position of the dominant (most frequently used) TSS, signal supporting that TSS and total signal in the cluster. The obtained clusters reflect context-specific promoterome and can be used as reference positions for genome-wide promoter-centred analyses, as a more precise and functionally relevant alternative to annotation. Furthermore, the downstream analyses described below provide additional layers of information for each promoter, allowing their classification and correlation of promoter features with other genome-wide data. Together with direct access to TSSs for numerous human and mouse cell and tissue types from FANTOM5 resource that can be easily included into the *CAGEr* workflow, it is a very powerful tool that can improve the resolution of any TSS-centred analysis.

**Table 1.** Summary of TSS clustering methods supported in *CAGEr*

Method	Main parameter	Level of supervision	Clusters
distclu	distance between neighbouring TSSs	semi data-driven	non-overlapping
paraclu	density of transcription initiation events	data-driven	overlapping (can be merged to non-overlapping)
custom	predefined genomic windows	user-defined	overlapping or non-overlapping

### Promoter width

**Figure 3.**
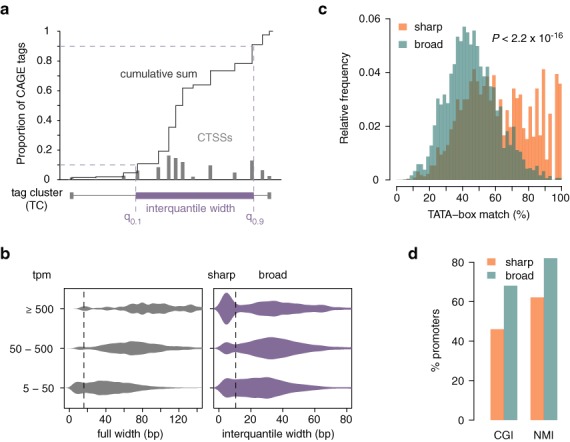
Promoter width. (**a**) Schematic representation of promoter width assessment using quantile positions of CAGE signal along the promoter. (**b**) Distribution of promoter width in adult mouse testis for three groups of promoters divided by expression (normalized CAGE tpm). Left panel shows the distribution of the full width from the most 5′ TSS to the most 3′ TSS in the promoter and right panel shows the interquantile width (distance between the positions of the 10^th^ and the 90^th^ percentile). Interquantile width accounts for local level of noise and provides a more robust measure of promoter width, allowing separation of sharp and broad promoters (dashed line). (**c**) Distribution of match (%) to TATA-box motif in the region −35 to −22 bp upstream of the dominant TSS in sharp and broad promoters. *P*-value of two-tailed Wilcoxon rank-sum test is shown. (**d**) Percentage of sharp and broad promoters that overlap CpG islands (CGI) and non-methylated islands (NMI; data from (24)).

Genome-wide mapping of TSSs with CAGE initially revealed two main types of promoters with respect to the number and distribution of TSSs: ‘sharp’ (also called ‘peaked’ or ‘focused’) promoters in which the majority of transcription starts at one clearly dominant TSS, and ‘broad’ (‘dispersed’) promoters with several commonly used TSS positions distributed along a wider region (2). This promoter feature is conserved across Metazoa and correlates with both underlying sequence and chromatin configuration as well as with function of the associated gene (reviewed in (12)). Thus, promoter width is a useful concept that can provide insight into the mode of gene regulation in a particular regulatory environment. For example, extensive use of sharp promoters might indicate that the transcription is directed by a factor bound at a fixed distance to the TSS, which poses spatial constraint on RNAPII positioning (21). In *CAGEr*, we provide a method for assessing promoter width based on cumulative distribution of CAGE signal along the promoter. Instead of using the full span of the TC, interquantile width is defined as spacing between the positions of the two quantiles of the total CAGE signal (q_low_ and q_up_; Figure 3a). That way only the central region containing more than (q_up_–q_low_) × 100% of CAGE tags is considered, which gives a more robust estimate of promoter width with respect to expression level. To facilitate data exploration, *CAGEr* produces tracks for visualization of interquantile width of individual promoters in a transcript-like representation (Supplementary Figure S3a). As demonstrated for the adult mouse testis sample, full length of the cluster is largely dependent on the absolute expression and the depth of sequencing, and with the increasing depth of recent sequencing technologies does not show the expected bimodal distribution in case of highly expressed promoters, giving the impression that the majority of those promoters are fairly broad (Figure 3b). On the other hand, interquantile width reveals that a substantial proportion of those promoters are actually sharp, as expected for highly expressed TATA-box associated promoters (Figure 3c). Thus, interquantile width accounts for local level of noise and brings the distribution of promoter width across different magnitudes of expression to the same scale, allowing easier separation of sharp and broad promoters (Figure 3b). The underlying difference between these two promoter classes is clearly evident in their association with TATA-box, which is mainly found in sharp promoters, and CpG islands and non-methylated regions, which more often overlap with broad promoters (Figure 3c, d). Thus, assessing interquantile width with *CAGEr* and plotting the distribution of promoter width in different samples gives and overview of the global usage of the different promoter types and hints at the predominant mode of regulation in a particular context (Supplementary Figure S3b). *CAGEr* workflow allows context-specific assignment of promoters into sharp or broad class by applying few simple commands (R code in Supplementary Methods), providing an additional layer of information that can be integrated into any promoter-centred analysis.

### Expression profiling

**Figure 4.**
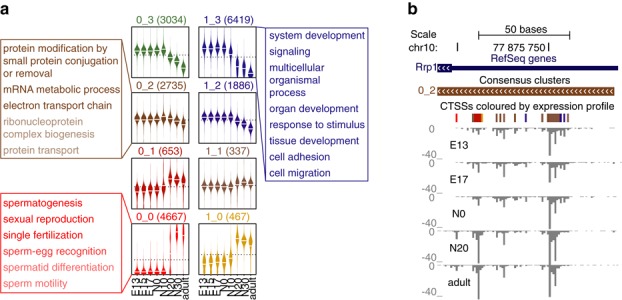
Promoter-centred expression profiling. (**a**) Self-organizing map clustering of promoter expression across eight mouse testis samples. Each box represents one cluster and the number of contained promoters is denoted above the box. Individual beanplots show distribution of scaled normalized expression for those promoters in different samples denoted on the x-axis. Gene ontology terms significantly enriched in selected clusters are shown in corresponding colours. (**b**) Example of a constitutively expressed promoter that contains TSSs with distinct expression dynamics. First track shows the span of the cluster (promoter) and is coloured according to its expression class (0_2) as shown in panel (a). Second track shows individual TSS positions with signal above 5 tpm, which are coloured according to their own expression class as shown in Supplementary Figure S5b.

*CAGEr* manipulates multiple CAGE samples at once and can address promoter dynamics across different contexts. To perform expression profiling at promoter level, TSS clusters from individual samples are first aggregated into a single set of consensus clusters, as shown schematically in Supplementary Figure S4. This produces more robust boundaries of the promoter region and captures all transcription initiation associated with a single gene. Promoters are then distributed into expression classes by applying one of the two commonly used unsupervised clustering algorithms: *k*-means or self-organizing maps (30) (SOM), which are invoked with single command in *CAGEr* (R code in Supplementary Methods). The resulting expression profiles are visualized using beanplots (31) and in the case of SOM they are organized into a two-dimensional map with the neighbouring clusters being more similar than the distant ones. An example of 2 × 4 SOM trained on a set of promoter expression values across mouse testis development time-course is shown in Figure 4a, which clearly separates promoters specific for the mature adult testis from the promoters active only in the earlier developmental stages. Different expression clusters are enriched for different gene ontology terms, reflecting the biological functions relevant in different stages of testis development (Figure 4a; Supplementary Table S1). Expression dynamics of individual promoters adds another layer of information for integrative analyses and can also be exported for visualization in the genome browser by colouring promoters according to their expression cluster (Figure 4b; Supplementary Figure S5a).

An analogous expression clustering can be performed on the level of individual TSS positions, which reveals similar expression profiles (Supplementary Figure S5b). Importantly, the expression patterns of individual TSSs within promoter region do not always correspond to the overall expression pattern of the promoter, suggesting dynamic changes in relative usage of TSSs across the time-course, as revealed by colouring them according to their expression profile (Figure 4b; Supplementary Figure S5c).

### Differential TSS usage and promoter shifting

**Figure 5.**
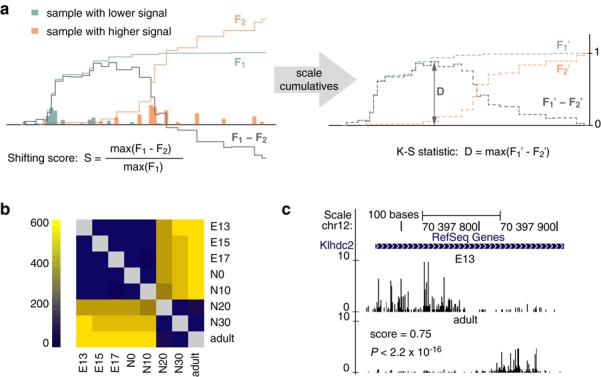
Differential TSS usage. (**a**) Schematics of differential TSS usage assessment. Distribution of TSSs and cumulative distribution of CAGE signal (F_1_ and F_2_) along single promoter in two different samples is shown in cyan and orange, respectively. Grey line shows the subtraction of the two cumulatives. The shifting score is calculated as a ratio of the maximal difference between the two cumulatives and the total CAGE signal at that promoter in the sample with lower signal (left panel). The cumulatives are scaled to the range between 0 and 1, and Kolmogorov–Smirnov (K–S) test is used to assess the significance of the difference between resulting empirical distribution functions (F_1_′ and F_2_′). Value of the K–S statistic (D) is illustrated by an arrow (right panel). (**b**) Number of promoters with significant differential TSS usage (K–S test, FDR ≤ 0.01) for all pair-wise comparisons of eight mouse testis samples. (**c**) Example of a shifting promoter detected using method shown in panel a, which demonstrates differential TSS usage between mouse embryonic (E13) and adult testis. Shifting score and corrected K–S *P*-value are denoted.

The discrepancy between the expression dynamics of the entire promoter and the contained individual TSSs indicates differential TSS usage across samples. This often results in spatial separation of TSS usage within a relatively narrow promoter region producing ‘shifting’ promoter patterns (21) (Figure 5c). *CAGEr* systematically detects such cases by comparing cumulative distributions of CAGE signal along the same consensus promoter region in two different (groups of) samples. Each individual promoter is scored for shifting as shown in Figure 5a. The resulting score can be interpreted as the proportion of transcription initiation in the sample with lower total expression that is shifted either upstream or downstream of the region used for initiation in the sample with the higher expression. For instance, the score of 0.4 means that at least 40% of the transcription in one sample is independent and happening outside of the region used to initiate transcription from the same promoter in the other sample. A set of promoters with shifting score above specified threshold between any two (groups of) samples can be easily obtained in *CAGEr* with only few simple commands (R code in Supplementary Methods) and can be further used to analyse features underlying differential TSS usage (21).

Shifting score reflects the degree of spatial separation in TSS usage within a promoter. However, it does not show the statistical significance of the observed difference. To address this, *CAGEr* tests the significance of the difference between the two cumulatives of the CAGE signal along the promoter using the Kolmogorov–Smirnov (K–S) test. For each promoter, the maximal difference between the two empirical distribution functions describing cumulative CAGE signal in the two different samples corresponds to the K–S statistic (arrow in Figure 5a), which is used to derive the probability that the two CAGE signals at that promoter are drawn from the same distribution (*P*-value). In addition to capturing clear spatial separation characterized by a high shifting score (Figure 5c), significant *P*-value also identifies more complex patterns of differential TSS usage intertwined within the same region, such as partial TSS gain or loss that leads to narrowing or broadening of the promoter (Supplementary Figure S6). By combining shifting score with K–S *P*-value, different types of differential TSS usage can be distinguished.

Applying this approach to a previously uncharacterized set of mouse testis CAGE samples revealed extensive promoter shifting detected mainly between early embryonic and adult testis (Figure 5b), and identified hundreds of promoters differentially used in the two regulatory environments (Supplementary Table S2). This switch in the promoter usage happened between the neonate days 10 and 20 (Figure 5b, Supplementary Figure S6) and corresponded to the transcriptional activation of a large set of genes involved in spermatogenesis (Figure 4a), suggesting major changes in the regulatory environment during spermatogenesis that might be driving promoter shifting. Once a reliable set of differentially used promoters is obtained, they can be further dissected and analysed to establish the underlying sequence and chromatin features directing TSS choice in different contexts (21).

### Resources of precise TSS data accessible through *CAGEr*

Several large collections of CAGE data have been published, including ENCODE data for multiple common human cell lines (19), and recent FANTOM5 collection covering vast majority of primary cells and tissues in human and mouse (11). Despite being a valuable resource of precise and context-specific TSSs, these data are not yet widely used, due to CAGE being less common than some other genome-wide experiments and due to a lack of comprehensive workflows that would integrate easy access to a user-friendly format of the data with methods for its processing and visualization. To address this, we have collected majority of previously published CAGE datasets into R data packages. These include numerous samples for common cell lines from ENCODE (19), for human and mouse tissues from previous FANTOM projects (8–10) and for zebrafish developmental time-course from our previous work (20) (Table 2). The most recent FANTOM5 collection (11) is too vast to distribute as a data package, so we have implemented direct query and retrieval of individual TSS sets for selected samples from the FANTOM5 web resource. All these resources are easily accessible with only a few commands in *CAGEr* and can be included directly into the provided workflow (R code examples in Supplementary Methods), greatly increasing the accessibility of precise TSS data for integrative analyses in R.

**Table 2.** Resources of CAGE-detected TSSs accessible directly from within *CAGEr*

Resource	Type	Organism	Sample type	Nr. samples	Reference
FANTOM5	online resource	human	cell lines, primary cells, tissues	988	(11)
		mouse		395	
FANTOM3 and 4	R data package	human	tissues, time-courses	100	(8–10)
		mouse		83	
ENCODE	R data package	human	cell lines	132	(19)
		fruit fly	whole embryo	1	(6)
Zebrafish development	R data package	zebrafish	developmental time-course	12	(20)

**Figure 6.**
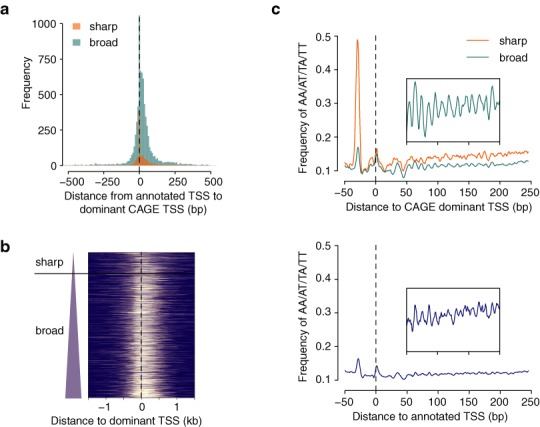
Comparison between annotated TSS and CAGE. (**a**) Distance between annotated RefSeq TSS and dominant TSS of the closest CAGE tag cluster in adult mouse testis. Promoters have been separated into sharp and broad class based on their interquantile width as shown in Figure 3b. (**b**) Non-methylated DNA signal (data from (24)) at promoters sorted by interquantile width and centred at CAGE dominant TSS. Broad promoters are associated with broader non-methylated regions and the level of non-methylation increases with promoter width. (**c**) Frequency of AA/AT/TA/TT dinucleotides around sharp and broad promoters centred at CAGE dominant TSS (top) or RefSeq annotated TSS (bottom). Magnified view of the signal in the region 50–200 bp downstream of the TSS is shown in the inset and demonstrates the 10 bp periodicity linked to nucleosome positioning (32) in broad promoters. Unlike RefSeq annotation, CAGE allows separation of sharp and broad promoters (Figure 3b) and adds precision into promoter-centred analysis revealing subtle sequence patterns in different classes of promoters.

Unlike annotations from RefSeq and Ensembl, which are still the commonly used reference for various promoter-centred analyses, CAGE data provides more precise and context-specific TSS information. This data is both of superior resolution and often significantly different from annotated TSS sets (Figure 6a), and provides additional layers of information about promoter width and architecture that can be integrated into analysis (Figure 6b, c). We believe that these precise and context-specific TSS data should be used instead of RefSeq and similar annotations wherever possible to increase the resolution and functional relevance of promoter-centred analyses. Precise TSSs can reveal spatial constraints and subtle patterns in sequence and chromatin features of promoters as demonstrated by the 10 bp periodicity in WW dinucleotide frequency starting ∼50 bp downstream of the dominant TSS in broad promoters in adult mouse testis and indicating intra-nucleosomal positioning signal (32), which is missed by using RefSeq annotation (Figure 6c).

## DISCUSSION

CAGE data represents resource of precise and context-specific TSSs widely applicable in various approaches, from computational genome-wide analyses to designing constructs for transgenesis. Here we introduced *CAGEr*, a comprehensive R/Bioconductor software package that implements various methods for CAGE data processing and promoterome mining and allows construction of a high-resolution, context-specific promoterome through a well-documented and user-friendly workflow. The package further introduces novel approaches for analysing promoter structure and dynamics, which provide additional layers of information, allowing classification of promoters and correlation of promoter features with other genome-wide data.

One of the key functionalities implemented in *CAGEr* is the robust assessment of promoter width—a feature that distinguishes different functional classes of promoters (2,6,12). The application and functional relevance of promoter interquantile width has been corroborated in several recent studies, which revealed different sequence signature and nucleosome positioning associated with sharp and broad promoters across numerous human and mouse cell types (11), as well as differential usage of these promoter types during zebrafish embryonic development (20). Furthermore, we have shown recently that promoter width is not an inherent property of the genomic locus, but is rather dependent on the regulatory context that drives the expression in the given cell type or condition, as demonstrated by the global change in the architecture of ubiquitously expressed promoters during maternal to zygotic transition in zebrafish (21). This highlights the need for the context-specific promoter width assessment.

Selection of individual TSSs within promoter region is context-dependent (16,21) and *CAGEr* can be used to detect differential promoter usage between different samples. In our recent study, we used the shifting score-based approach to successfully decouple two independent transcription initiation codes that overlap on thousands of core promoters and produce different readouts from the same promoter during maternal to zygotic transition in zebrafish (21). Here we introduce an implementation of this approach expanded with a method for assessing statistical significance and demonstrate its applicability to mouse testis developmental CAGE data revealing extensive differential TSS usage between mouse embryonic and adult testis. This enables further exploration of sequence, chromatin, transcription factor binding or any other feature that might be driving differential TSS choice.

Most importantly, *CAGEr* and accompanying data packages provide easy access to majority of publicly available CAGE datasets for numerous samples from several organisms in the form that can be easily integrated with other genome-wide data. These include large TSS collections for human and mouse derived from ENCODE (19) and FANTOM (11) CAGE data, as well as smaller TSS datasets for zebrafish (20) and fruit fly (6). Direct access to these precise TSS data that can be easily included into the *CAGEr* workflow combined with the comprehensive promoter mining functionality provided in the package, present a very powerful tool that can improve the resolution of any TSS-centred analysis. Precise TSSs are crucial for investigating spatial constraints between transcription initiation and sequence motifs or epigenetic modifications in core promoters (11,21) and are particularly important when analysing high-resolution data such as bisulphite sequencing or single nucleotide polymorphisms.

## AVAILABILITY

*CAGEr* package is free open-source software distributed through Bioconductor and both source code and executables are available at http://www.bioconductor.org/packages/release/bioc/html/CAGEr.html. *FANTOM3and4* data package is also distributed through Bioconductor at http://www.bioconductor.org/packages/release/data/experiment/html/FANTOM3and4CAGE.html. *ENCODEprojectCAGE* and *ZebrafishDevelopmentalCAGE* data packages are freely available from authors’ website at http://promshift.genereg.net/CAGEr/PackageSource/. All packages are fully documented and accompanied by detailed user guides available at http://promshift.genereg.net/CAGEr/Vignettes/.

## SUPPLEMENTARY DATA

Supplementary Data are available at NAR Online.

SUPPLEMENTARY DATA
