# Supplementary material for: CAGEr: precise TSS data retrieval and high-resolution promoterome mining for integrative analyses
Source: Nucleic Acids Res. 2015 Feb 4;43(8):e51. doi: 10.1093/nar/gkv054 (PMC4417143; doi:10.1093/nar/gkv054)
Supplement: SUPPLEMENTARY DATA [file supp_43_8_e51__index.html]

CAGEr: precise TSS data retrieval and high-resolution promoterome mining for integrative analyses — CAGEr: precise TSS data retrieval and high-resolution promoterome mining for integrative analyses — SUPPLEMENTARY DATA 

# *CAGEr*: precise TSS data retrieval and high-resolution promoterome mining for integrative analyses

## SUPPLEMENTARY DATA

**Files in this Data Supplement:**

- SUPPLEMENTARY DATA
- SUPPLEMENTARY DATA
- SUPPLEMENTARY DATA
