# Supplementary material for: CAGEr: precise TSS data retrieval and high-resolution promoterome mining for integrative analyses
Source: Nucleic Acids Res. 2015 Feb 4;43(8):e51. doi: 10.1093/nar/gkv054 (PMC4417143; doi:10.1093/nar/gkv054)
Supplement: SUPPLEMENTARY DATA [file supp_gkv054_nar-03623-met-k-2014-File008.pdf]

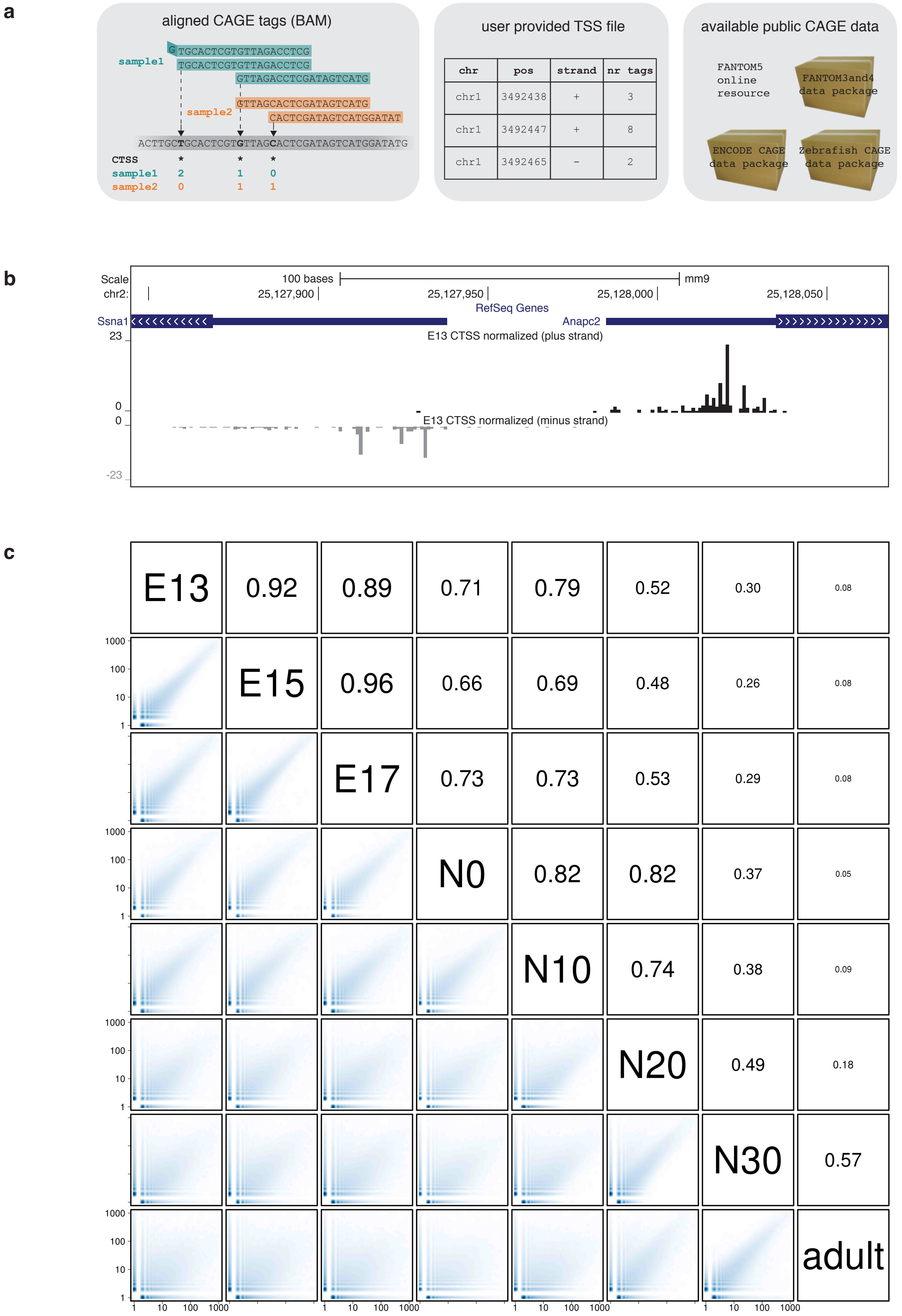


**Supplementary Figure S1.** Overview of CAGE samples imported in *CAGEr*. (**a**) Three types of input supported in *CAGEr*. (**b**) Strand-specific single base-pair resolution TSSs visualised in UCSC genome browser using tracks produced by *CAGEr*. TSSs on negative strand are shown as facing downwards. The height of each bar corresponds to normalised tag per million values and reflects relaive expression from that TSS. (**c**) Scatter plots of CAGE tag counts per CTSS for pair-wise comparisons of 8 mouse testis development samples are shown in the lower left triangle. Numbers in the upper right triangle denote Pearson's correlation coefficients. Sample names are denoted on the diagonal.

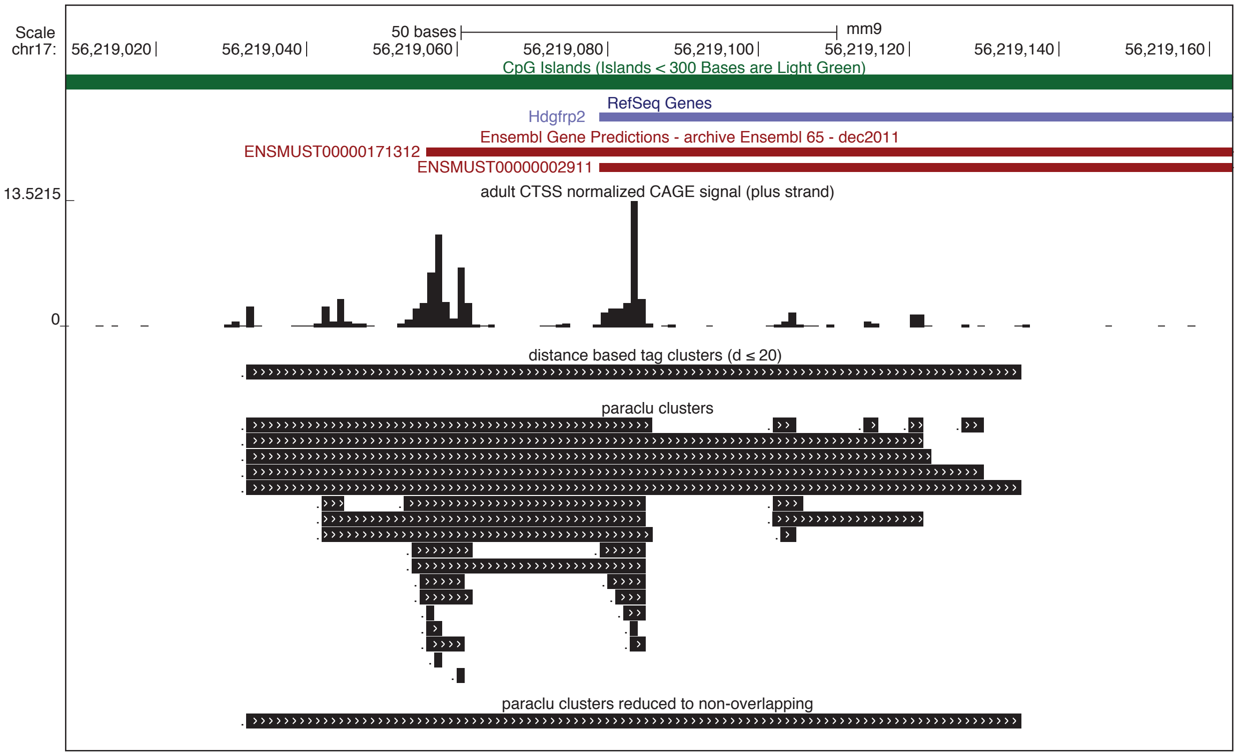


**Supplementary Figure S2**. TSS clustering into tag clusters (TC). Clusters produced using two different methods are shown for *Hdgfrp2* promoter region in adult mouse testis. Distance based clusters were created by joining together neighbouring TSSs less than 20 bp apart (top). Paraclu clusters were created by applying parametric clustering algorithm and restricting cluster length to 500bp, which produces overlapping clusters and reflects hierarchical organisation (middle). Paraclu-derived clusters can be merged to produce non-overlapping set of clusters (bottom).

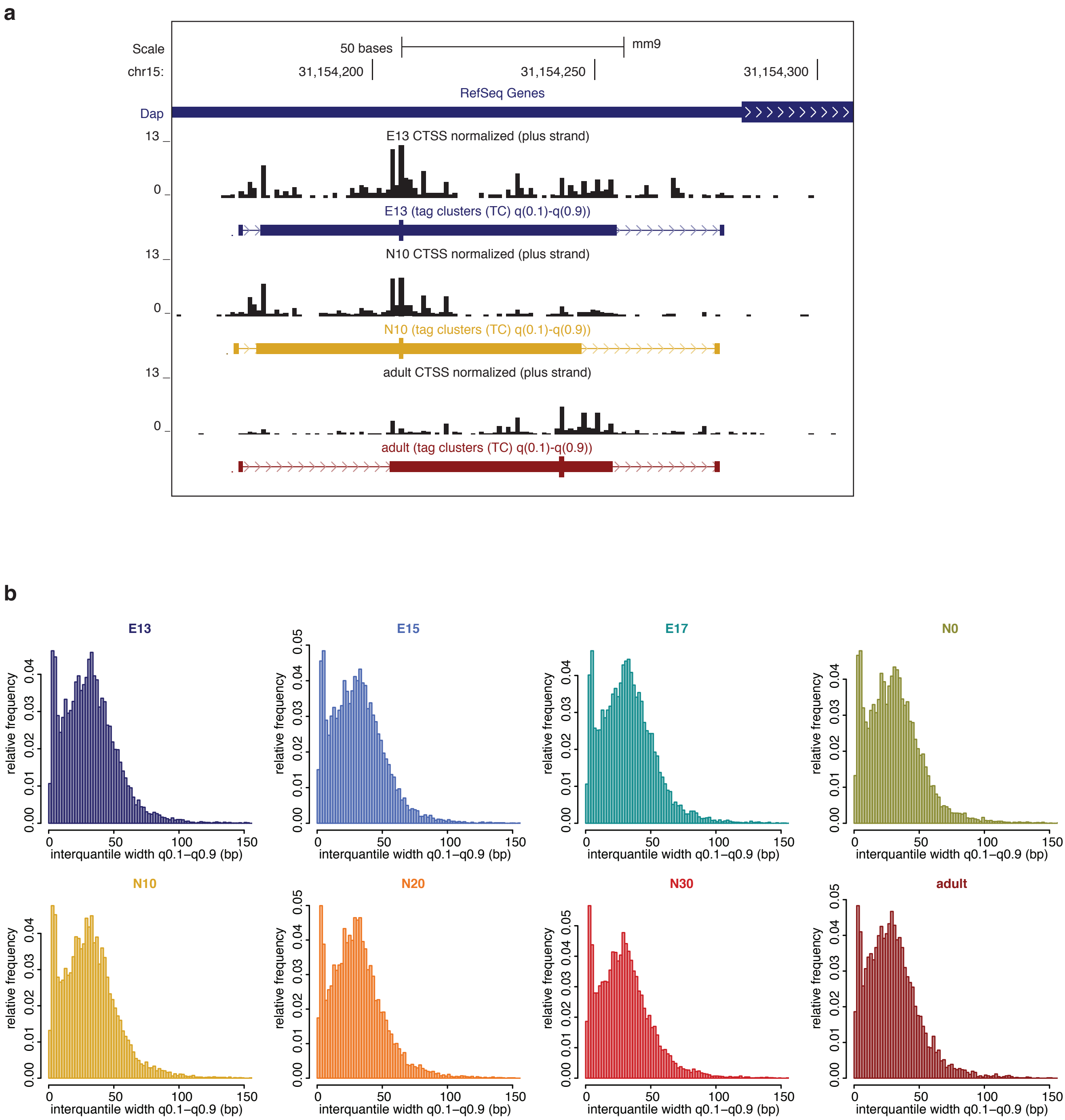


**Supplementary Figure S3**. Promoter width (**a**) Visualisation of TC interquantile width and position of dominant TSS in the genome browser using tracks exported by *CAGEr*. TCs in three different samples are shown, demonstrating differences in promoter width and preferred TSS position within the same promoter. (**b**) Histograms showing distribution of interquantile width of tag clusters (TCs) in each individual mouse testis sample created with *CAGEr*. Only TCs expressed above 5 tpm in given sample were included and interquantile width was calculated as distance between positions of 10$^{th}$ (q0.1) and 90$^{th}$ (q0.9) percentile of CAGE signal.

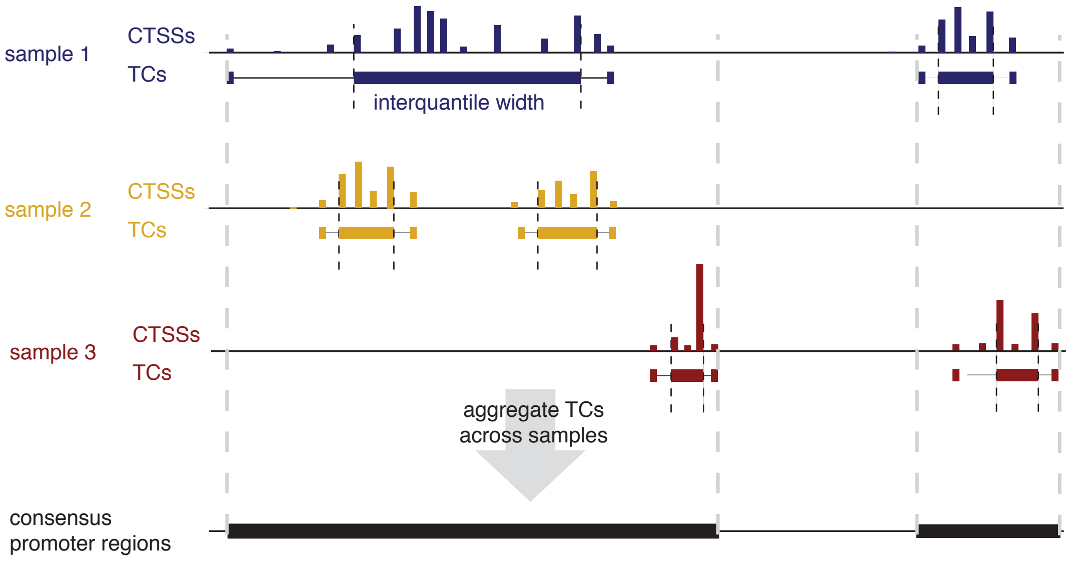


**Supplementary Figure S4**. Consensus promoter regions are established as follows. (1) TSS clusters (TCs) are constructed for each sample individually by applying any of the available clustering methods. (2) Boundaries of each TC are set by the user to be either the full span of the cluster or the interquantile width (filled block). (3) TCs from individual samples are aggregated so that TCs that directly overlap or whose boundaries are within defined proximity of each other end up in the same promoter region. This establishes the boundaries of the promoters and creates a single set of consensus promoter regions. Resulting promoters are used for expression profiling and for detecting promoter shifting and differential TSS usage.

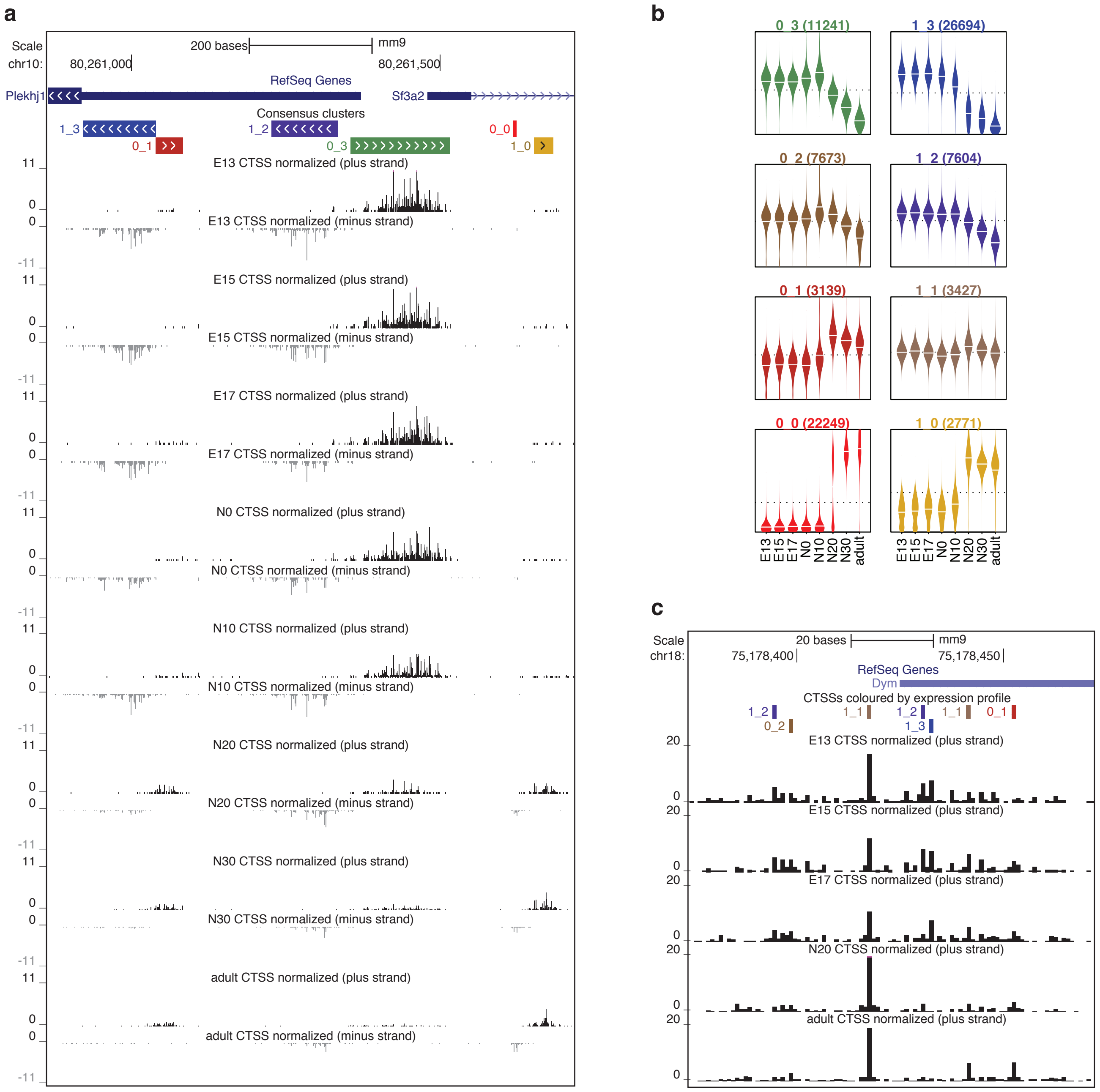


**Supplementary Figure S5.** Visualisation of expression profiles. (**a**) After expression profiling is done and promoters are distributed into different expression classes, a data track for visualisation of expression patterns can be exported using *CAGEr*. Each promoter is coloured and named according to the expression class to which it belongs. Shown here is an example of a complex locus with multiple promoters with clearly different expression dynamics. Promoters are coloured according to expression classes shown in Figure 4a derived from self-organizing map clustering. (**b**) Self-organizing map clustering of individual TSSs based on expression across 8 mouse testis samples. Only TSSs supported with more than 5 tpm in at least one sample were selected. Each box represents one cluster and the number of contained TSSs is denoted above the box. Individual beanplots show distribution of scaled normalized expression for those TSSs in different samples denoted on the x-axis. (**c**) Visualisation of expression profiles of inidividual TSSs in the genome browser. Each TSS is coloured and named according to the expression class to which it belongs as shown in panel b. TSSs with different expression dynamics are present in the same promoter.

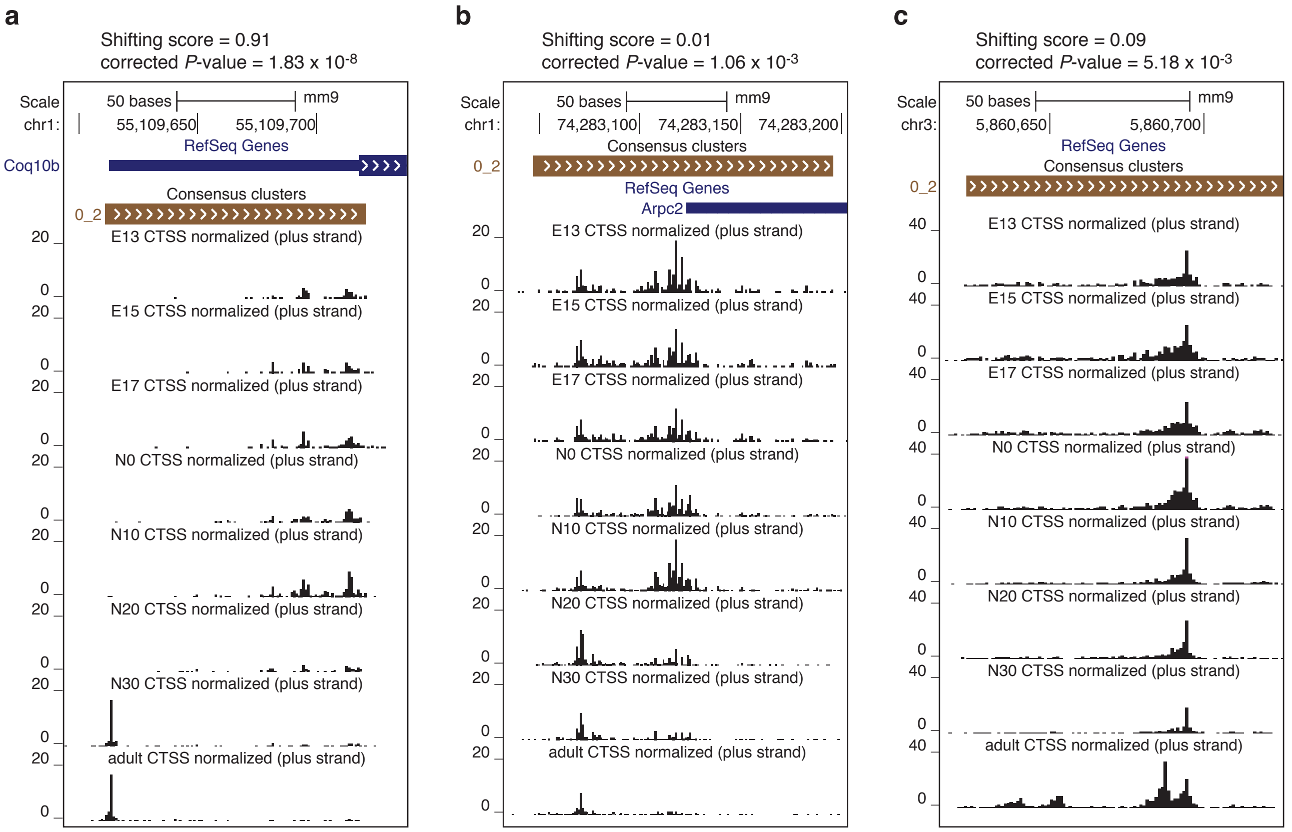


**Supplementary Figure S6**. Various types of differential promoter usage detected by *CAGEr* between embryonic and adult mouse testis (**a**) Complete shifting of TSSs within promoter region of *Coq10b* gene. (**b**) Partial loss of TSSs and narrowing of the promoter region of *Arpc2* gene. (**c**) Partial gain of TSSs within an unannotated promoter.
