# Supplementary material for: CAGEr: precise TSS data retrieval and high-resolution promoterome mining for integrative analyses
Source: Nucleic Acids Res. 2015 Feb 4;43(8):e51. doi: 10.1093/nar/gkv054 (PMC4417143; doi:10.1093/nar/gkv054)
Supplement: SUPPLEMENTARY DATA [file supp_gkv054_nar-03623-met-k-2014-File009.pdf]

Supplementary Methods

1. *CAGEr* Bioconductor package vignette..1

2. R code for mouse testis CAGE data analysis..40

3. R code examples for accessing public CAGE data resources..................43

CAGEr: an R package for CAGE (Cap Analysis of Gene Expression) data analysis and promoterome mining

Vanja Haberle *

November 28, 2014

Contents

1 Introduction **2**

2 Input data for *CAGEr* **5**

3 Getting started **5**

4 *CAGEr* workflow **6**
4.1 Specifying input files 6
4.2 Creating `CAGEset` object 6
4.3 Reading in the data 8
4.4 Correlation between samples 9
4.5 Normalization 10
4.6 Exporting CAGE signal to bedGraph 12
4.7 CTSS clustering 13
4.8 Promoter width 14
4.9 Creating consensus promoters across samples 17
4.10 Expression profiling 19
4.11 Shifting promoters 21

5 Accessing public CAGE datasets **24**
5.1 Available CAGE data resources 24
5.1.1 FANTOM5 24
5.1.2 FANTOM3 and 4 25
5.1.3 ENCODE cell lines 25
5.1.4 Zebrafish developmental timecourse 26

*Department of Biology, University of Bergen, Bergen, Norway

5.2 Importing public TSS data for manipulation in *CAGEr* 26
5.2.1 FANTOM5 human and mouse samples 26
5.2.2 *FANTOM3and4CAGE* data package 31
5.2.3 *ENCODEprojectCAGE* data package 34
5.2.4 *ZebrafishDevelopmentalCAGE* data package 35

6 Session Info **36**

1 Introduction

This document briefly describes how to use the package *CAGEr*. *CAGEr* is a Bioconductor-compliant R package designed to manipulate, analyse and visualise Cap Analysis of Gene Expression (CAGE) sequencing data. CAGE (Kodzius et al. (2006)) is a high-throughput method for transcriptome analysis that utilizes "cap-trapping" (Carninci et al. (1996)), a technique based on the biotinylation of the 7-methylguanosine cap of Pol II transcripts, to pulldown the 5'-complete cDNAs reversely transcribed from the captured transcripts. A linker sequence is ligated to the 5' end of the cDNA and a specific restriction enzyme is used to cleave off a short fragment from the 5' end. Resulting fragments are then amplified and sequenced using massive parallel high-throughput sequencing technology, which results in a large number of short sequenced tags that can be mapped back to the referent genome to infer the exact position of the transcription start sites (TSSs) used for transcription of captured RNAs (Figure 1). Number of CAGE tags supporting each TSS gives the information on relative frequency of its usage and can be used as a measure of expression from that specific TSS. Thus, CAGE provides information on two aspects of capped transcriptome: genome-wide 1bp-resolution map of transcription start sites and transcript expression levels. This information can be used for various analyses, from 5' centered expression profiling (Takahashi et al. (2012)) to studying promoter architecture (Carninci et al. (2006)).

CAGE samples derived from various organisms (genomes) can be analysed by *CAGEr* and the only limitation is the availability of the referent genome as a *BSgenome* package in case when raw mapped CAGE tags are processed. *CAGEr* provides a comprehensive workflow that starts from mapped CAGE tags and includes reconstruction of TSSs and promoters and their visualisation, as well as more specialized downstream analyses like promoter width, expression profiling and differential TSS usage. It can use both Binary Sequence Alignment Map (BAM) files of aligned CAGE tags or files with genomic locations of TSSs and number of supporting CAGE tags as input. If BAM files are provided *CAGEr* constructs TSSs from aligned CAGE tags and counts the number of tags supporting each TSS, while allowing filtering out low-quality tags and removing technology-specific bias. It further performs normalization of raw CAGE tag count, clustering of TSSs into tag clusters (TC) and their aggregation across multiple CAGE experiments into promoters to construct the promoterome. Various methods for normalization and clustering of TSSs are supported. Exporting data into different types of track

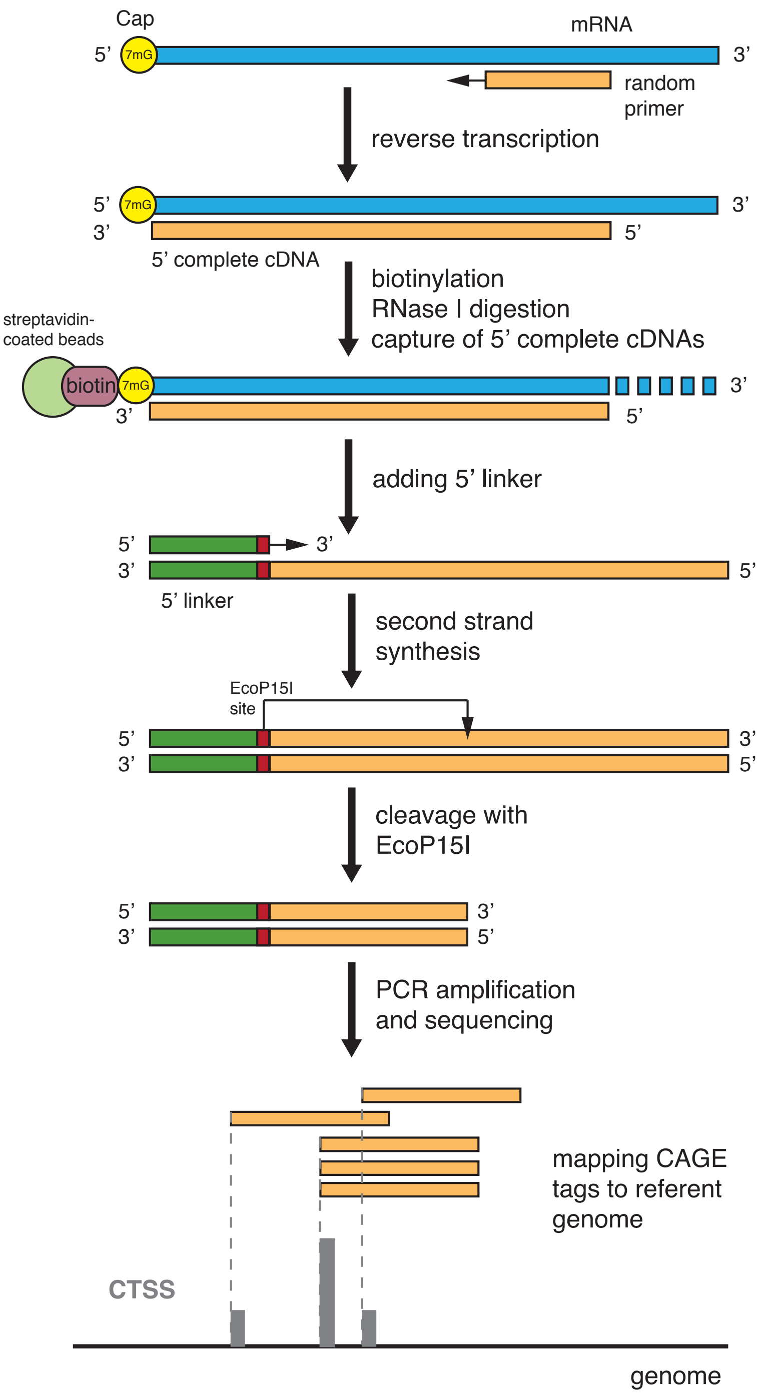

Figure 1: Overview of CAGE experiment

files allows various visualisations of TSSs and clusters (promoters) in the UCSC Genome Browser, which facilitate generation of hypotheses. *CAGEr* manipulates multiple CAGE experiments at once and performs analyses across datasets, including expression profiling and detection of differential TSS usage (promoter shifting). Multicore option for parallel processing is supported on Unix-like platforms, which significantly reduces computing time.

Here are some of the functionalities provided in this package:

- Reading in multiple CAGE datasets from various sources; user provided BAM or TSS input files, public CAGE datasets from accompanying data package.
- Correcting systematic G nucleotide addition bias at the 5' end of CAGE tags.
- Plotting pairwise scatter plots, calculating correlation between datasets and merging datasets.
- Normalizing raw CAGE tag count: simple tag per million (tpm) or power-law based normalization (Balwierz et al. (2009)).
- Clustering individual TSSs into tag clusters (TCs) and aggregating clusters across multiple CAGE datasets to create a set of consensus promoters.
- Making bedGraph or BED files of individual TSSs or clusters for visualisation in the genome browser.
- Expression clustering of individual TSSs or consensus promoters into distinct expression profiles using common clustering algorithms.
- Calculating promoter width based on the cumulative distribution of CAGE signal along the promoter.
- Scoring and statistically testing differential TSS usage (promoter shifting) and detecting promoters that shift between two samples.

A data package *FANTOM3and4CAGE* is accompanying this package. It contains all of the up-to-date publicly available CAGE data produced by FANTOM, a genome regulation-oriented international consortium, and provides a valuable resource of genome-wide TSSs in various tissue/cell types in human and mouse that can be used directly in R. Section 5 in this vignette describes how these public datasets can be included into a workflow provided by *CAGEr*. See the vignette of the *FANTOM3and4CAGE* package for the list of available CAGE datasets.

2 Input data for *CAGEr*

CAGEr package supports three types of CAGE data input:

1. **Sequenced CAGE tags mapped to the genome**: BAM (Binary Sequence Alignment Map) files of sequenced CAGE tags aligned to the referent genome.
2. **CAGE detected TSSs (CTSSs)**: tab separated files with genomic coordinates of TSSs and number of tags supporting each TSS. The file should not contain a header and the data must be organized in four columns:
   - name of the chromosome: names must match the names of chromosomes in the corresponding *BSgenome* package
   - 1-based coordinate of the TSS on the chromosome
   - genomic strand: should be either + or -
   - number of CAGE tags supporting that TSS
3. **Publicly available CAGE datasets from R data package**: A data package *FANTOM3and4CAGE* containing CAGE data produced by FANTOM consortium is accompanying this package. Selected subset of these data can be used as input for *CAGEr*.

3 Getting started

To load the *CAGEr* package into your R envirnoment type:

```
> library(CAGEr)
```

In this tutorial we will be using data from zebrafish (*Danio rerio*) that was mapped to the danRer7 assembly of the genome. Therefore, the corresponding genome package *BSgenome.Drerio.UCSC.danRer7* has to be installed and available to load by typing:

```
> library(BSgenome.Drerio.UCSC.danRer7)
```

In case the data is mapped to a genome that is not readily available through *BSgenome* package (not in the list returned by `available.genomes()` function), a custom *BSgenome* package has to be build and installed first. (See the vignette within the *BSgenome* package for instructions on how to build a custom genome package). The built genome should then be loaded by calling `library()` and the `genomeName` argument should be set to the name of the build genome package when creating a `CAGEset` object (see the section *Creating* `CAGEset` *object* below).

4 *CAGEr* workflow

4.1 Specifying input files

The subset of zebrafish (*Danio rerio*) developmental time-series CAGE data generated by (Nepal et al. (2013)) will be used for the demonstration of the workflow and the provided functionalities. Files with genomic coordinates of TSSs detected by CAGE in 4 zebrafish developmental stages are included in this package in the `extdata` subdirectory. The files contain TSSs from a part of chromosome 17 (26,000,000-46,000,000), and there are two files for one of the developmental stages (two independent replicas). The data in files is organized in four tab separated columns as described above in the section about input data formats. First we have to define paths to the input files:

```
> inputDir <- system.file("extdata", package = "CAGEr")
> pathsToInputFiles <- list.files(inputDir, full.names = TRUE)
> basename(pathsToInputFiles)
```

```
[1] "Zf.30p.dome.chr17.ctss"
[2] "Zf.high.chr17.ctss"
[3] "Zf.prim6.rep1.chr17.ctss"
[4] "Zf.prim6.rep2.chr17.ctss"
[5] "Zf.unfertilized.egg.chr17.ctss"
```

4.2 Creating `CAGEset` object

We start the workflow by creating a `CAGEset` object, which is a container for storing CAGE datasets and all the results that will be generated by applying specific functions. `CAGEset` is created by providing name of the referent genome, paths to input files, type of input files and labels of individual CAGE datasets (samples):

```
> myCAGEset <- new("CAGEset", genomeName = "BSgenome.Drerio.UCSC.danRer7",
+                  inputFiles = pathsToInputFiles, inputFilesType = "ctss",
+                  sampleLabels = c("zf_30p_dome", "zf_high",
+                  "zf_prim6_rep1", "zf_prim6_rep2", "zf_unfertilized_egg"))
```

To display the created object type:

```
> myCAGEset
```

```
S4 Object of class CAGEset

========================================
Input data information
========================================
```

```
Reference genome (organism): BSgenome.Drerio.UCSC.danRer7
Input file type: ctss
Input file names: /private/var/folders/ql/vxgzffss11s0lqshr1kz08m40000gn/T/RtmpgvY86N/R
Sample labels: zf_30p_dome, zf_high, zf_prim6_rep1, zf_prim6_rep2, zf_unfertilized_egg
=======================================
CTSS information
=======================================
CTSS chromosome:
CTSS position:
CTSS strand:
Tag count:
Normalized tpm:
=======================================
Tag cluster (TC) information
=======================================
CTSS clustering method:
Number of TCs per sample:
=======================================
Consensus cluster information
=======================================
Number of consensus clusters:
Consensus cluster chromosome:
Consensus cluster start:
Consensus cluster end:
Consensus cluster strand:
Normalized tpm:
=======================================
Expression profiling
=======================================
Expression clustering method:
Expression clusters for consensus clusters:
=======================================
Promoter shifting
=======================================
GroupX:
GroupY:
Shifting scores:
KS p-values (FDR adjusted):
```

The supplied information can be seen in the `Input data information` section, whereas all other slots are still empty since no data has been read yet and no analysis conducted.

4.3 Reading in the data

To read in the data from the provided files we use the following function:

```
> getCTSS(myCAGEset)
```

This function reads in the data from the provided files in the order they were specified in the `inputFiles` argument. It creates a single set of all TSSs detected across all input datasets and a table with counts of CAGE tags supporting each TSS in every dataset. Genomic coordinates of all TSSs and numbers of supporting CAGE tags in every input sample can be retrieved by typing:

```
> ctss <- CTSStagCount(myCAGEset)
> head(ctss)

    chr      pos strand zf_30p_dome zf_high zf_prim6_rep1
1 chr17 26027430      +           0       0             1
2 chr17 26050540      +           0       0             0
3 chr17 26068225      -           1       0             0
4 chr17 26068227      -           1       0             0
5 chr17 26068233      -           1       0             0
6 chr17 26074127      -           2       0             0
  zf_prim6_rep2 zf_unfertilized_egg
1             0                   0
2             0                   1
3             0                   0
4             0                   0
5             0                   0
6             0                   2
```

Note that the samples are ordered in the way they were supplied when creating the `CAGEset` object and will be presented in that order in all the results and plots. To check sample labels and their ordering type:

```
> sampleLabels(myCAGEset)

            #FF0000FF             #CCFF00FF
        "zf_30p_dome"             "zf_high"
            #00FF66FF             #0066FFFF
      "zf_prim6_rep1"       "zf_prim6_rep2"
            #CC00FFFF
"zf_unfertilized_egg"
```

In addition, a colour is assigned to each sample, which is consistently used to depict that sample in all the plots. By default a rainbow palette of colours is used and the hexadecimal format of the assigned colours can be seen as names attribute of sample labels shown above. The colours can be changed to taste at any point in the workflow using `setColors` function.

4.4 Correlation between samples

After the data has been read we can start exploring the data by looking at the correlation between the samples. The `plotCorrelation` function will plot pairwise scatter plots of CAGE tag count per TSS and calculate correlation between all possible pairs of samples. A tag count threshold can be set, so that only TSSs with tag count above the threshold (either in one or both samples) are considered when calculating correlation. Three different correlation measures are supported: Pearson's, Spearman's and Kendall's correlation coefficients.

```
> corr.m <- plotCorrelation(myCAGEset, samples = "all", method = "pearson")
```

The command above will create a PNG file with pairwise scatter plots and calculated correlation coefficients (Figure 2), and will return a matrix with correlation coefficients. Note that the correlation is calculated using raw tag counts on a linear scale, however, the scatter plots are plotted on a logarithmic scale for the convenience. Figure 2 shows that early developmental stages correlate very well with each other, whereas their correlation with later prim6 stage is lower. There is also a very good correlation between the two replicas for prim6 developmental stage.

Based on calculated correlation we might want to merge and/or rearrange some of the datasets. To rearrange the samples in the temporal order of the zebrafish development (unfertilized egg -> high -> 30 percent dome -> prim6) and to merge the two replicas for the prim6 developmental stage we use the `mergeSamples` function:

```
> mergeSamples(myCAGEset, mergeIndex = c(3,2,4,4,1),
+                         mergedSampleLabels = c("zf_unfertilized_egg",
+                         "zf_high", "zf_30p_dome", "zf_prim6"))
```

The `mergeIndex` argument controls which samples will be merged and how the final dataset will be ordered. Samples labeled by the same number (in our case samples three and four) will be merged together by summing number of CAGE tags per TSS. The final set of samples will be ordered in the ascending order of values provided in `mergeIndex` and will be labeled by the labels provided in the `mergedSampleLabels` argument. Note that `mergeSamples` function resets all slots with results of downstream analyses, so in case there were any results in the `CAGEset` object prior to merging, they will be removed.

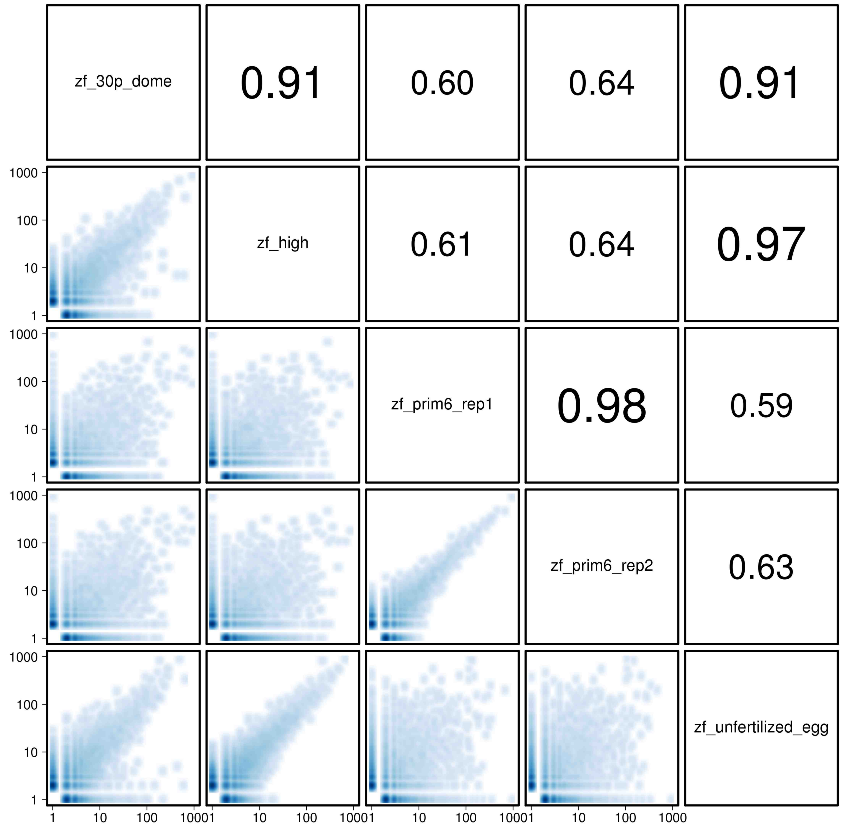

Figure 2: Correlation of raw CAGE tag counts per TSS

4.5 Normalization

Library sizes (number of total sequenced tags) of individual experiments differ, thus normalization is required to make them comparable. The `librarySizes` function returns the total number of CAGE tags in each sample:

```
> librarySizes(myCAGEset)

zf_unfertilized_egg              zf_high          zf_30p_dome
              56140                45910                41814
           zf_prim6
              69000
```

CAGEr package supports both simple tags per million normalization and power-law based normalization. It has been shown that many CAGE datasets follow a power-law distribution (Balwierz et al. (2009)). Plotting the number of CAGE tags (X-axis) against the number of TSSs that are supported by <= of that number of tags (Y-axis) results in a distribution that can be approximated by a power-law. On a log-log scale this reverse cumulative distribution will manifest as a monotonically decreasing linear

function, which can be defined as
`y = -1 * alpha * x + beta`
and is fully determined by the slope `alpha` and total number of tags T (which together with `alpha` determines the value of `beta`).
To check whether our CAGE datasets follow power-law distribution and in which range of values, we can use the `plotReverseCumulatives` function:

```
> plotReverseCumulatives(myCAGEset, fitInRange = c(5, 1000), onePlot = TRUE)
```

This will create a PDF file with reverse cumulative plots (Figure 3) in your working directory. In addition, a power-law distribution will be fitted to each reverse cumulative using values in the specified range (denoted with dashed lines in Figure 3) and the value of alpha will be reported for each sample (shown in the brackets in the Figure 3 legend). The plots can help in choosing the optimal parameters for power-law based normalization. We can see that the reverse cumulative distributions look similar and follow the power-law in the central part of the CAGE tag counts values with a slope between -1.1 and -1.3. Thus, we choose a range from 5 to 1000 tags to fit a power-law, and we normalize all samples to a referent power-law distribution with a total of 50,000 tags and slope of -1.2 (`alpha = 1.2`). (Note that since this example dataset contains only data from one part of chromosome 17 and the total number of tags is very small, we normalize to a referent distribution with a similarly small number of tags. When analyzing full datasets it is reasonable to set total number of tags for referent distribution to one million to get normalized tags per million values.)

To perform normalization we pass these parameters to the `normalizeTagCount` function:

```
> normalizeTagCount(myCAGEset, method = "powerLaw",
+                   fitInRange = c(5, 1000), alpha = 1.2, T = 5*10^4)
```

The normalization is performed as described in (Balwierz et al. (2009)):

1. Power-law is fitted to the reverse cumulative distribution in the specified range of CAGE tags values to each sample separately.

2. A referent power-law distribution is defined based on the provided `alpha` (slope in the log-log representation) and `T` (total number of tags) parameters. Setting `T` to 1 million results in normalized tags per million (tpm) values.

3. Every sample is normalized to the defined referent distribution, *i.e.* given the parameters that approximate its own power-law distribution it is calculated how many tags would each TSS have in the referent power-law distribution.

In addition to the two provided normalization methods, a pass-through option ”none” can be set as `method` parameter to keep using raw tag counts in all downstream steps. Note that `normalizeTagCount` function has to be applied to `CAGEset` object before

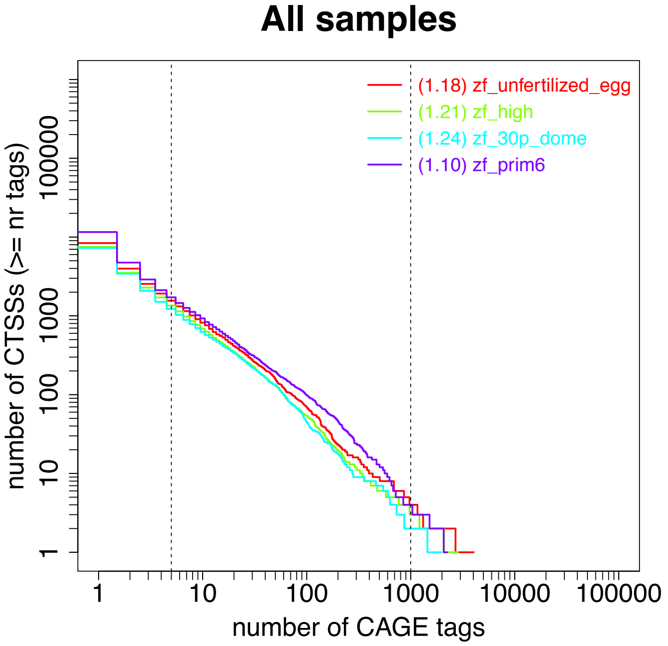

Figure 3: Reverse cumulative distribution of CAGE tags

moving to next steps. Thus, in order to keep using raw tag counts run the function with `method="none"`. In that case, all results and parameters in the further steps that would normally refer to normalized CAGE signal (denoted as tpm), will actually be raw tag counts.

4.6 Exporting CAGE signal to bedGraph

CAGE data can be visualized in the genomic context by exporting raw or normalized CAGE tag counts to a bedGraph file and uploading the file to a genome browser. Positions of TSSs and tag counts supporting them are exported using `exportCTSStoBedGraph` function:

```
> exportCTSStoBedGraph(myCAGEset, values = "normalized", oneFile = TRUE)
```

This will produce a single bedGraph file with multiple annotated tracks that can be directly visualized as custom tracks in the genome browser (Figure 4). There are two tracks per sample; one for TSSs on the plus strand and the other for the minus strand. Values for TSSs on minus strand are shown as negative and are pointing downwards in the browser.

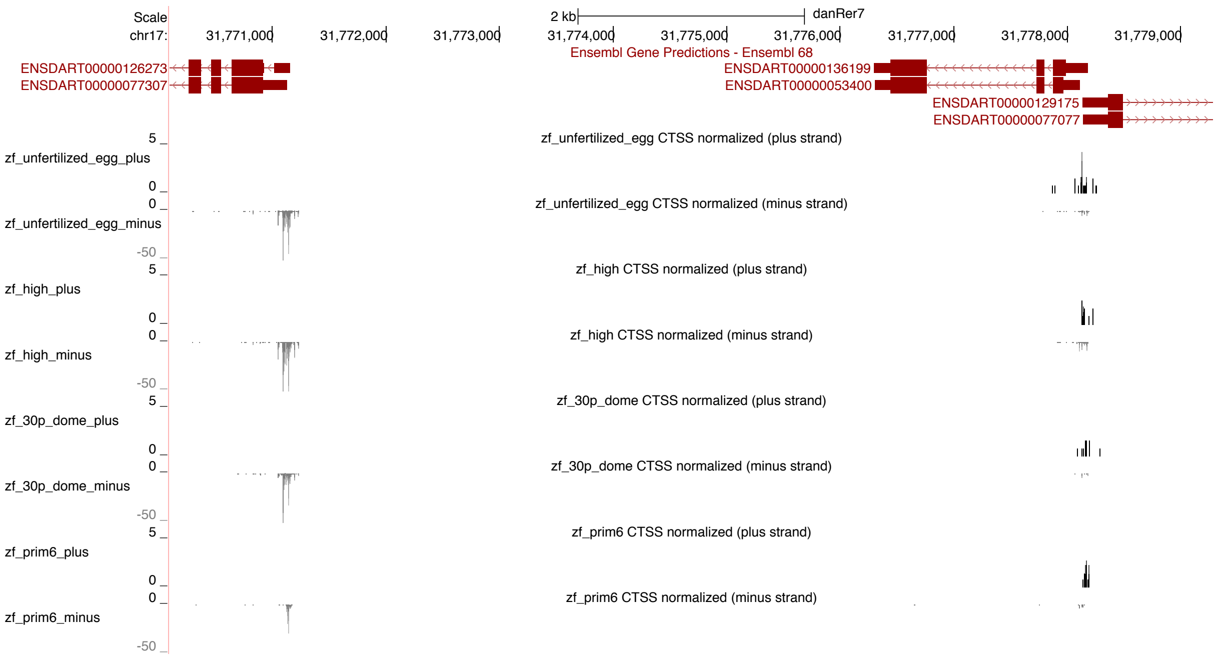

Figure 4: CAGE data bedGraph track visualized in the UCSC Genome Browser

4.7 CTSS clustering

Transcription start sites are found in the promoter region of a gene and reflect the transcriptional activity of that promoter (Figure 4). TSSs in the close proximity of each other give rise to a functionally equivalent set of transcripts and are likely regulated by the same promoter elements. Thus, TSSs can be spatially clustered into larger transcriptional units, called tag clusters (TCs) that correspond to individual promoters. *CAGEr* supports three methods for spatial clustering of TSSs along the genome, two "ab initio" methods driven by the data itself as well as assigning TSSs to predefined genomic regions:

1. simple distance-based clustering in which two neighbouring TSSs are joined together if they are closer than some specified distance

2. parametric clustering of data attached to sequences based on the density of the signal (Frith et al. (2007), http://www.cbrc.jp/paraclu/)

3. counting TSSs and their signal in a set of user supplied genomic regions (*e.g.* annotation derived promoter regions or other regions of interest)

These functionalities are provided in the `clusterCTSS` function, which accepts additional arguments for controlling which CTSSs will be included in the clustering as well as for refining the final set of tag clusters.

We will perform a simple distance-based clustering using 20bp as a maximal allowed

distance between two neighbouring TSSs. Prior to clustering we will filter out low-fidelity TSSs - the ones supported by less than 2 normalized tag counts in all of the samples.

```
> clusterCTSS(object = myCAGEset, threshold = 1, thresholdIsTpm = TRUE,
+                    nrPassThreshold = 1, method = "distclu", maxDist = 20,
+                    removeSingletons = TRUE, keepSingletonsAbove = 5)
```

Our final set of tag clusters will not include singletons (clusters with only one TSS), unless the normalized signal is above 5, *i.e.* it is a reasonably supported TSS. The (clusterCTSS) function creates a set of clusters for each sample separately; for each cluster it returns the genomic coordinates, counts the number of TSSs within the cluster, determines the position of the most frequently used (dominant) TSS, calculates the total CAGE signal within the cluster and CAGE signal supporting the dominant TSS only. We can extract tag clusters for a desired sample from the `CAGEset` object by calling `tagClusters` function:

```
> tc <- tagClusters(myCAGEset, sample = "zf_unfertilized_egg")
> head(tc)

  cluster   chr    start      end strand nr_ctss
1       1 chr17 26453631 26453708      +      12
2       2 chr17 26564507 26564610      +      24
3       3 chr17 26595636 26595793      +      35
4       4 chr17 26596032 26596091      +       9
5       5 chr17 26596117 26596127      +       4
6       6 chr17 26596149 26596175      +       5
  dominant_ctss       tpm tpm.dominant_ctss
1      26453667  26.97094          8.250137
2      26564585 128.63720         29.283272
3      26595750 216.99944        100.970037
4      26596070  10.42000          3.216864
5      26596118  12.19946          5.741802
6      26596153  10.96552          3.850167
```

4.8 Promoter width

Genome-wide mapping of TSSs using CAGE has initially revealed two major classes of promoters in mammals (Carninci et al. (2006)), with respect to the number and distribution of TSSs within the promoter. They have been further correlated with differences in the underlying sequence and the functional classes of the genes they regulate, as well as the organization of the chromatin around them. These are:

- "broad" promoters with multiple TSSs characterized by a high GC content and overlap with a CpG island, which are associated with widely expressed or developmentally regulated genes
- "sharp" promoters with one dominant TSS often associated with a TATA-box at a fixed upstream distance, which often regulate tissue-specific transcription

Thus, the width of the promoter is an important characteristic that distinguishes different functional classes of promoters. *CAGEr* package analyzes promoter width across all samples present in the `CAGEset` object. It defines promoter width by taking into account both the positions and the CAGE signal at TSSs along the tag cluster, thus making it more robust with respect to total expression and local level of noise at the promoter. Width of every tag cluster is calculated as following:

1. Cumulative distribution of CAGE signal along the cluster is calculated.
2. Positions of two selected quantiles are determined. At the 5' end the position of the "lower" quantile `qLow` is determined, which is defined as the point that divides the cluster into two parts, such that the 5' part contains `< qLow * 100%` of the CAGE signal of that cluster. Accordingly, position of the "upper" quantile `qUp` is determined near the 3' end, which is defined as the point that divides the cluster into two parts such that the 5' part contains `>= qUp * 100%` of the CAGE signal of that cluster.
3. Promoter width is defined as the distance (in base pairs) between the two quantiles. This **interquantile width** marks the central part of the cluster that contains `>= (qUp - qLow) * 100%` of the CAGE signal.

The procedure is schematically shown in Figure 5.

Required computations are done using `cumulativeCTSSdistribution` and `quantilePositions` functions, which calculate cumulative distribution for every tag cluster in each of the samples and determine the positions of selected quantiles, respectively:

```
> cumulativeCTSSdistribution(myCAGEset, clusters = "tagClusters")
> quantilePositions(myCAGEset, clusters = "tagClusters", qLow = 0.1, qUp = 0.9)
```

Tag clusters and their interquantile width can be retrieved by calling `tagClusters` function:

```
> tc <- tagClusters(myCAGEset, sample = "zf_unfertilized_egg",
+                   returnInterquantileWidth = TRUE,  qLow = 0.1, qUp = 0.9)
> head(tc)

  cluster   chr    start      end strand nr_ctss
1       1 chr17 26453631 26453708      +      12
2       2 chr17 26564507 26564610      +      24
```

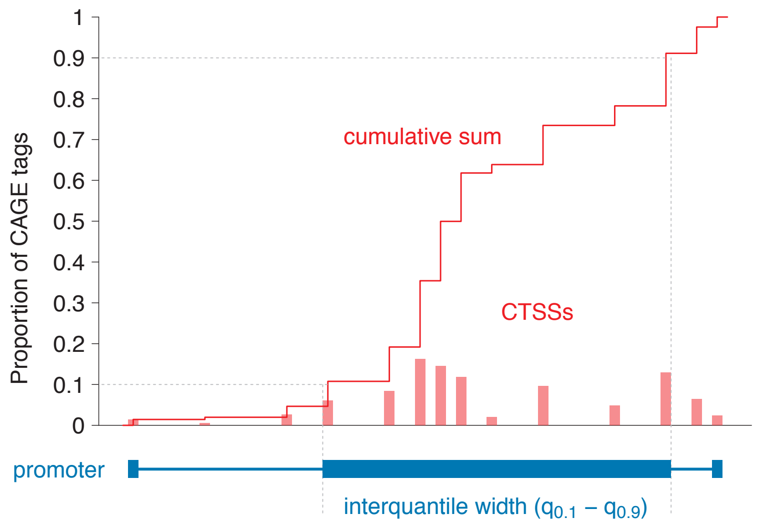

Figure 5: Cumulative distribution of CAGE signal and definition of interquantile width

```
3       3 chr17 26595636 26595793      +      35
4       4 chr17 26596032 26596091      +       9
5       5 chr17 26596117 26596127      +       4
6       6 chr17 26596149 26596175      +       5
  dominant_ctss       tpm tpm.dominant_ctss    q_0.1
1      26453667  26.97094          8.250137 26453667
2      26564585 128.63720         29.283272 26564524
3      26595750 216.99944        100.970037 26595673
4      26596070  10.42000          3.216864 26596033
5      26596118  12.19946          5.741802 26596118
6      26596153  10.96552          3.850167 26596151
     q_0.9 interquantile_width
1 26453703                  37
2 26564588                  65
3 26595750                  78
4 26596082                  50
5 26596127                  10
6 26596160                  10
```

Interquantile width can also be visualized in a gene-like representation in the UCSC genome browser by exporting the data into a BED file:

```
> exportToBed(object = myCAGEset, what = "tagClusters",
+                    qLow = 0.1, qUp = 0.9, oneFile = TRUE)
```

In this gene-like representation (Figure 6), the oriented line shows the full span of the cluster, filled block marks the interquantile width and a single base-pair thick block

denotes the position of the dominant TSS.

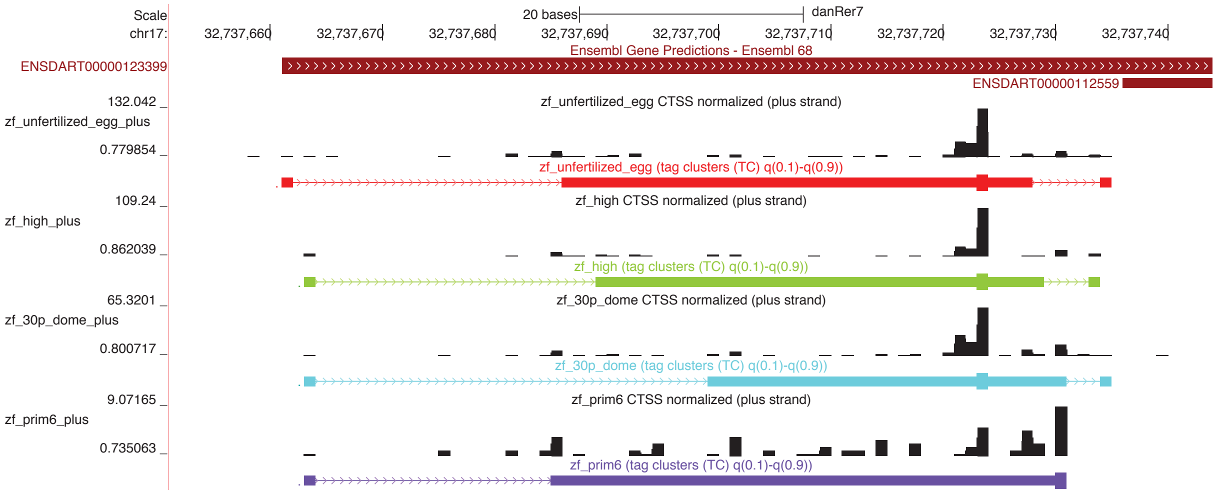

Figure 6: Tag clusters visualization in the genome browser

Once the cumulative distributions and the positions of quantiles have been calculated, the histograms of interquantile width can be plotted to globally compare the promoter width across different samples (Figure 7):

```
> plotInterquantileWidth(myCAGEset, clusters = "tagClusters",
+                        tpmThreshold = 3, qLow = 0.1, qUp = 0.9)
```

Significant difference in the promoter width might indicate global differences in the modes of gene regulation between the two samples. The histograms can also help in choosing an appropriate width threshold for separating sharp and broad promoters.

4.9 Creating consensus promoters across samples

Tag clusters are created for each sample individually and they are often sample-specific, thus can be present in one sample but absent in another. In addition, in many cases tag clusters do not coincide perfectly within the same promoter region, or there might be two clusters in one sample and only one larger in the other. To be able to compare genome-wide transcriptional activity across samples and to perform expression profiling, a single set of consensus clusters has to be created. This is done using `aggregateTagClusters` function, which aggregates tag clusters from all samples into a single set of non-overlapping consensus clusters:

```
> aggregateTagClusters(myCAGEset, tpmThreshold = 5,
+                        qLow = 0.1, qUp = 0.9, maxDist = 100)
```

Tag clusters can be aggregated using their full span (from start to end) or using positions of previously calculated quantiles as their boundaries. Only tag clusters above given

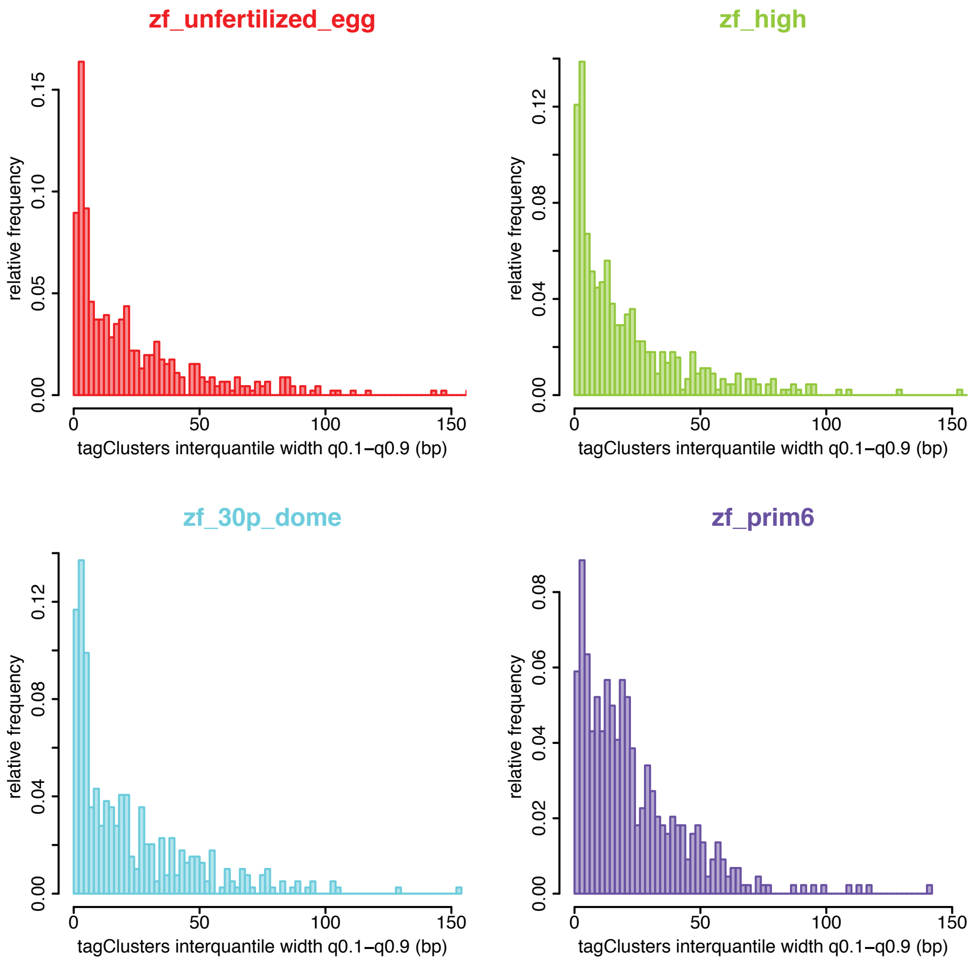

Figure 7: Distribution of promoter interquantile width

threshold will be selected and two clusters will be aggregated together if they are `<=` `maxDist` apart. Final set of consensus clusters can be retrieved by:

```
> consensusCl <- consensusClusters(myCAGEset)
> head(consensusCl)

  consensus.cluster   chr    start      end strand
1                 1 chr17 26453631 26453737      +
2                 2 chr17 26564507 26564611      +
3                 3 chr17 26595636 26595805      +
4                 4 chr17 26596032 26596339      +
5                 5 chr17 26645154 26645516      +
6                 6 chr17 26651962 26652050      +
        tpm
```

```
1  185.9678
2  318.3590
3 1017.8475
4  307.5127
5 2844.4560
6   71.0783
```

Analysis of promoter width can be performed for consensus clusters as well, using the `cumulativeCTSSdistribution`, `quantilePositions` and `plotInterquantileWidth` functions as described above for the tag clusters, but by setting the `clusters` parameter to `"consensusClusters"`.

4.10 Expression profiling

Since CAGE signal reflects level of transcription from a given TSS or promoter it can be used for 5' centered expression profiling. Expression clustering can be done at level of individual CTSSs or at level of entire promoters (consensus clusteres). In the former case, feature vector containing log transformed and scaled normalized CAGE signal at individual TSS across multiple samples is used as input for clustering algorithm, whereas in the latter case CAGE signal within the entire consensus cluster is used. *CAGEr* package supports two unsupervised clustering algorithms: kmeans and self-organizing maps (SOM). Both algorithms require to specify number of clusters in advance.
We will perform expression clustering at the level of entire promoter using SOM algorithm and applying it only to promoters with normalized CAGE signal $>=$ *15* in at least one sample.

```
> getExpressionProfiles(myCAGEset, what = "consensusClusters", tpmThreshold = 10,
+                 nrPassThreshold = 1, method = "som", xDim = 4, yDim = 2)
```

Distribution of expression across samples for 8 clusters returned by SOM (4 x 2 map) can be visualized using `plotExpressionProfiles` function as shown in Figure 8:

```
> plotExpressionProfiles(myCAGEset, what = "consensusClusters")
```

Each cluster is shown in different color and is marked by its label and the number of elements (promoters) in the cluster. We can extract promoters belonging to a specific cluster by typing:

```
> class3_1 <- extractExpressionClass(myCAGEset,
+                 what = "consensusClusters", which = "3_1")
> head(class3_1)

   consensus.cluster   chr    start      end strand
17                17 chr17 27293524 27293647      +
```

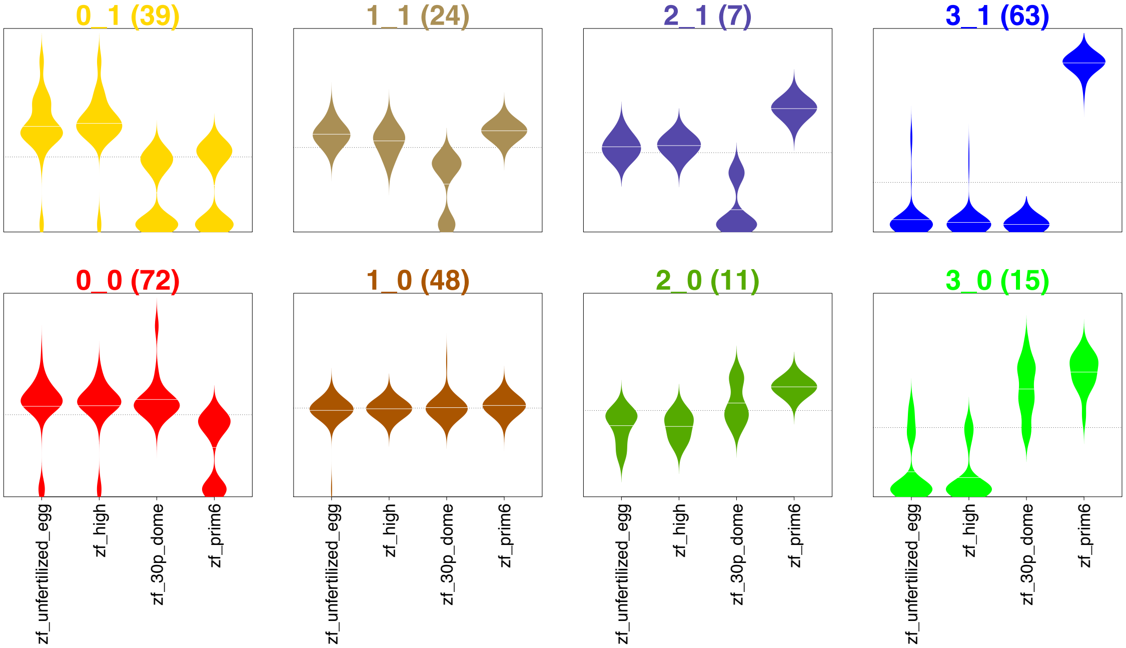

Figure 8: Expression clusters

```
36                 36 chr17 28740675 28740728      +
39                 39 chr17 29335651 29335661      +
40                 40 chr17 29378660 29378702      +
42                 42 chr17 29404735 29404741      +
46                 46 chr17 30507619 30507641      +
         tpm zf_unfertilized_egg   zf_high zf_30p_dome
17 157.31373             0.00000  0.000000           0
36  49.48524             0.00000  5.072922           0
39 234.10350            23.65617 13.216882           0
40  11.99385             0.00000  0.000000           0
42  11.49493             0.00000  0.000000           0
46  18.65917             0.00000  0.000000           0
    zf_prim6 expression_class
17 157.31373              3_1
36  44.41232              3_1
39 197.23045              3_1
40  11.99385              3_1
42  11.49493              3_1
46  18.65917              3_1
```

Consensus clusters and information on their expression profile can be exported to a BED file, which allows visualization of the promoters in the genome browser colored in the color of the expression cluster they belong to (Figure 9):

```
> exportToBed(myCAGEset, what = "consensusClusters",
+                      colorByExpressionProfile = TRUE)
```

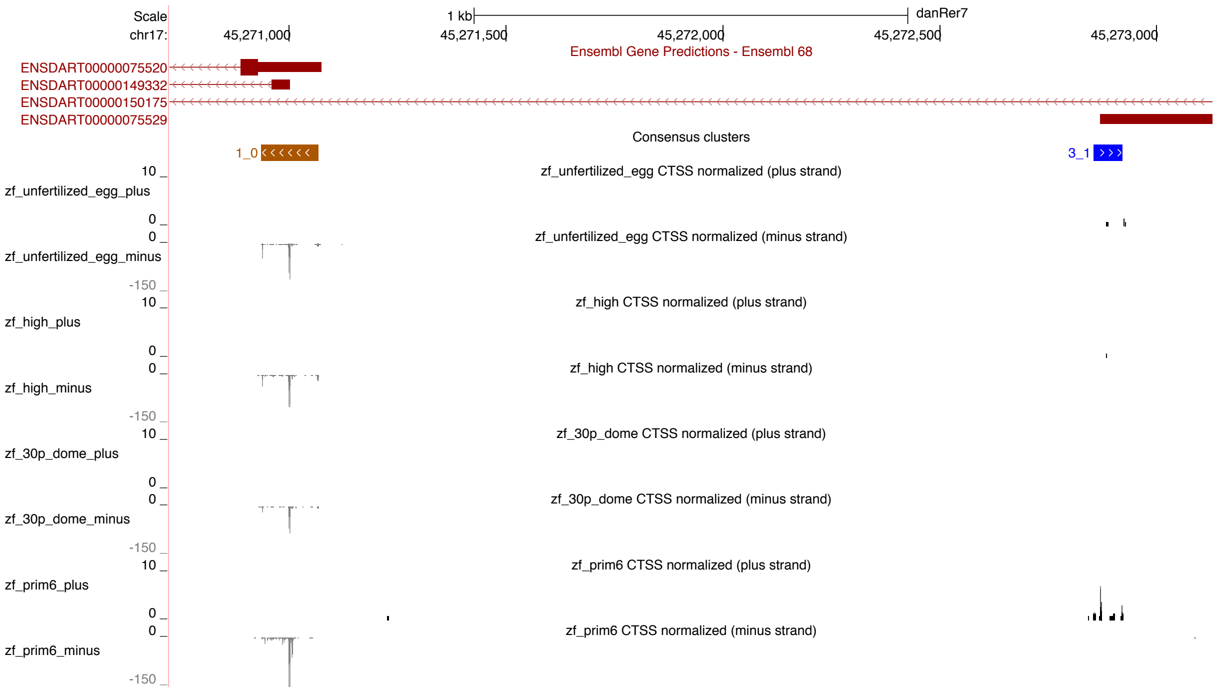

Figure 9: Consensus clusters colored by expression profile in the genome browser

Expression profiling of individual TSSs is done using the same procedure as described above for consensus clusters, only by setting `what` parameter to `"CTSS"` in all of the functions.

4.11 Shifting promoters

As shown in Figure 6, TSSs within the same promoter region can be used differently in different samples. Thus, although the overall transcription level from a promoter does not change between the samples, the differential usage of TSSs or "promoter shifting" may indicate changes in the regulation of transcription from that promoter, which cannot be detected by expression profiling. To detect this promoter shifting, a method described in Haberle et al. (2014) has been implemented in *CAGEr*. Shifting can be detected between two individual samples or between two groups of samples. In the latter case, samples are first merged into groups and then compared in the same way as two individual samples. For all promoters a shifting score is calculated based on the difference in the cumulative

distribution of CAGE signal along that promoter in the two samples. In addition, a more general assessment of differential TSS usage is obtained by performing Kolmogorov-Smirnov test on the cumulative distributions of CAGE signal, as described below. Thus, prior to shifting score calculation and statistical testing, we have to calculate cumulative distribution along all consensus clusters:

```
> cumulativeCTSSdistribution(myCAGEset, clusters = "consensusClusters")
```

Next, we calculate a shifting score and P-value of Kolmogorov-Smirnov test for all promoters comparing two specified samples:

```
> scoreShift(myCAGEset, groupX = "zf_unfertilized_egg", groupY = "zf_prim6",
+                    testKS = TRUE, useTpmKS = FALSE)
```

This function will calculate shifting score as illustrated in Figure 10. Values of shifting score are in range between -Inf and 1. Positive values can be interpreted as the proportion of transcription initiation in the sample with lower expression that is happening "outside" (either upstream or downstream) of the region used for transcription initiation in the other sample. In contrast, negative values indicate no physical separation, *i.e.* the region used for transcription initiation in the sample with lower expression is completely contained within the region used for transcription initiation in the other sample. Thus, shifting score detects only the degree of upstream or downstream shifting, but does not detect more general changes in TSS rearrangement in the region, *e.g.* narrowing or broadening of the region used for transcription.
To assess any general change in the TSS usage within the promoter region, a two-sample Kolmogorov-Smirnov (K-S) test on cumulative sums of CAGE signal along the consensus cluster is performed. Cumulative sums in both samples are scaled to range between 0 and 1 and are considered to be empirical cumulative distribution functions (ECDF) reflecting sampling of TSS positions during transcription initiation. K-S test is performed to assess whether the two underlying probability distributions differ. To obtain P-value (*i.e.* the level at which the null-hypothesis can be rejected), sample sizes that generated the ECDFs are required, in addition to actual K-S statistics calculated from ECDFs. These are derived either from raw tag counts, *i.e.* exact number of times each TSS in the cluster was sampled during sequencing (when `useTpmKS = FALSE`), or from normalized tpm values (when `useTpmKS = TRUE`). P-values obtained from K-S tests are further corrected for multiple testing using Benjamini and Hochenberg (BH) method and for each P-value a corresponding false-discovery rate (FDR) is also reported.

We can select a subset of promoters with shifting score and/or FDR above specified threshold:

```
> shifting.promoters <- getShiftingPromoters(myCAGEset,
+                    tpmThreshold = 5, scoreThreshold = 0.6,
+                    fdrThreshold = 0.01)
> head(shifting.promoters)
```

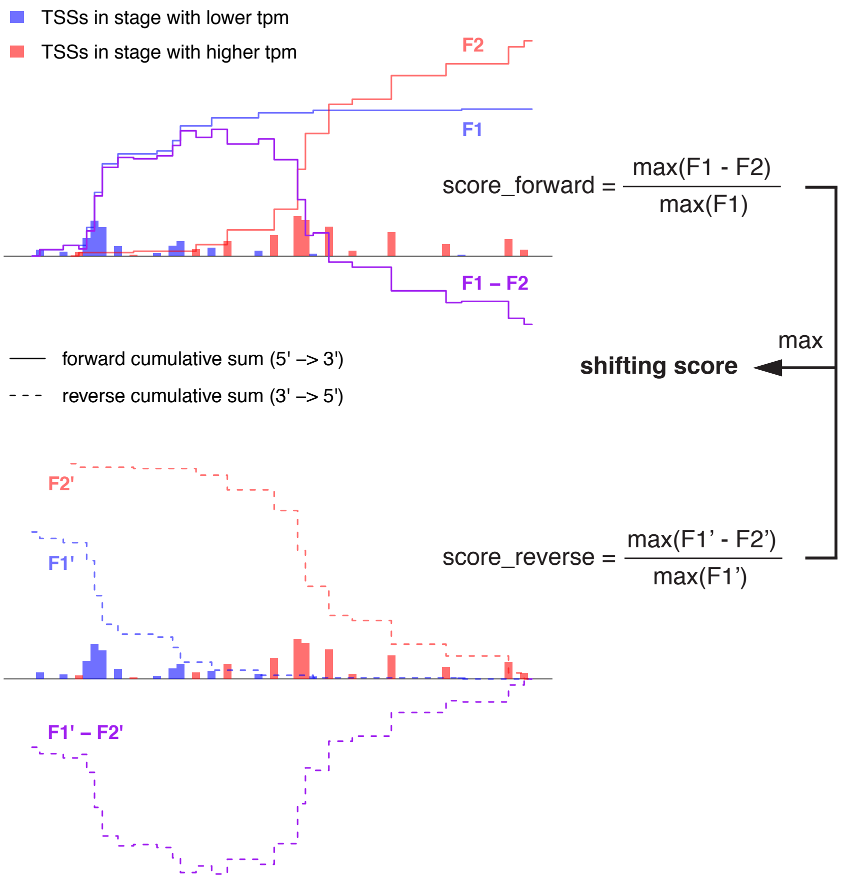

Figure 10: Calculation of shifting score

```
  consensus.cluster   chr    start      end strand
1                 3 chr17 26595636 26595805      +
2                86 chr17 33502377 33502477      +
3                90 chr17 33581353 33581440      +
4               117 chr17 37383273 37383399      +
5               118 chr17 37395387 37395504      +
6               141 chr17 39957522 39957582      +
  shifting.score groupX.pos groupY.pos groupX.tpm
1      0.6659092   26595750   26595709  216.99944
2      0.6541964   33502454   33502378   15.94950
3      0.6027427   33581374   33581407   22.59123
4      0.7452116   37383277   37383375   14.72790
5      0.6311793   37395410   37395468   61.07977
```

```
6      0.6971956   39957572   39957550   11.69832
  groupY.tpm    pvalue.KS       fdr.KS
1 258.321380 0.000000e+00 0.000000e+00
2   9.369801 1.350306e-05 2.591966e-05
3  15.107430 1.044059e-05 2.075689e-05
4  47.982965 4.894196e-12 1.634662e-11
5 134.501645 0.000000e+00 0.000000e+00
6   6.454123 1.656264e-03 2.659578e-03
```

The `getShiftingPromoters` function returns genomic coordinates, shifting score and P-value (FDR) of the promoters, as well as the value of CAGE signal and position of the dominant TSS in the two compared (groups of) samples. Figure 11 shows the difference in the CAGE signal between the two compared samples for one of the selected high-scoring shifting promoters.

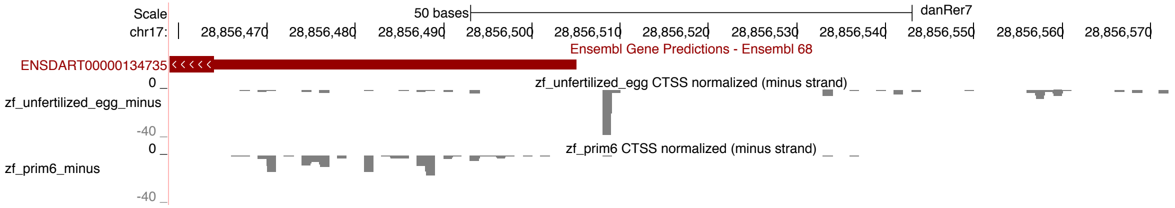

Figure 11: Example of shifting promoter

5 Accessing public CAGE datasets

5.1 Available CAGE data resources

There are several large collections of CAGE data available that provide single base-pair resolution TSSs for numerous human and mouse primary cells, cell lines and tissues. Together with several minor datasets for other model organisms (*Drosophila melanogaster*, *Danio rerio*) they are a valuable resource that provides cell/tissue type and developmental stage specific TSSs essential for any type of promoter centred analysis. By enabling direct and user-friendly import of TSSs for selected samples into R, *CAGEr* facilitates the integration of these precise TSS data with other genomic data types. Each of the available CAGE data resources accessible from within *CAGEr* is explained in more detail further below.

5.1.1 FANTOM5

FANTOM consortium provides single base-pair resolution TSS data for numerous human and mouse primary cells, cell lines and tissues. The main FANTOM5 publication

(Consortium (2014)) released ~1000 human and ~400 mouse CAGE samples that cover the vast majority of human primary cell types, mouse developmental tissues and a large number of commonly used cell lines. These data is available from FANTOM web resource at http://fantom.gsc.riken.jp/5/data/ in the form of TSS files with genomic coordinates and number of tags mapping to each TSS detected by CAGE. The list of all available samples for both human and mouse (as presented in the Supplementary Table 1 of the publication) has been included in *CAGEr* to facilitate browsing, searching and selecting samples of interest. TSSs for selected samples are then fetched directly form the web resource and imported into a `CAGEset` object enabling their further manipulation with *CAGEr*.

5.1.2 FANTOM3 and 4

Previous FANTOM projects (3 and 4) (Consortium (2005); Faulkner et al. (2009); Suzuki et al. (2009)) produced CAGE datasets for multiple human and mouse tissues as well as several timecourses, including differentiation of a THP-1 human myeloid leukemia cell line. All this TSS data has been grouped into datasets by the organism and tissue of origin and has been collected into an R data package named *FANTOM3and4CAGE*, which is available from Bioconductor (http://www.bioconductor.org/packages/release/data/experiment/html/F The vignette accompanying the package provides information on available datasets and lists of samples. When the data package is installed, *CAGEr* can import the TSSs for selected samples directly into a `CAGEset` object for further manipulation.

5.1.3 ENCODE cell lines

ENCODE consortium produced CAGE data for common human cell lines (Djebali et al. (2012)), which were used by ENCODE for various other types of genome-wide analyses. The advantage of this dataset is that it enables the integration of precise TSSs from a specific cell line with many other genome-wide data types provided by ENCODE for the same cell line. However, the format of CAGE data provided by ENCODE at UCSC (http://genome.ucsc.edu/ENCODE/dataMatrix/encodeDataMatrixHuman.html) includes only raw mapped CAGE tags and their coverage along the genome, and coordinates of enriched genomic regions (peaks), which do not take advantage of the single base-pair resolution provided by CAGE. To address this, we have used the raw CAGE tags to derive single base-pair resolution TSSs and collected them into an R data package named *ENCODEprojectCAGE*. This data package is available for download from CAGEr web site at http://promshift.genereg.net/CAGEr and includes TSSs for 36 different cell lines fractionated by cellular compartment. The vignette accompanying the package provides information on available datasets and lists of individual samples. Once the package has been downloaded and installed, *CAGEr* can access it to import TSS data for selected subset of samples for further manipulation and integration.

5.1.4 Zebrafish developmental timecourse

Precise TSSs are also available for zebrafish (*Danio Rerio*) from CAGE data published by Nepal *et al.* (Nepal et al. (2013)). The timecourse covering early embryonic development of zebrafish includes 12 developmental stages. The TSS data has been collected into an R data package named *ZebrafishDevelopmentalCAGE*, which is available for download from CAGEr web site at http://promshift.genereg.net/CAGEr. As with other data packages mentioned above, once the package is installed *CAGEr* can use it to import stage-specific single base pair TSSs into a `CAGEset` object.

5.2 Importing public TSS data for manipulation in *CAGEr*

The data from above mentioned resources can be imported into a `CAGEset` object using the `importPublicData()` function. The `importPublicData()` function has four arguments: `source`, `dataset`, `group` and `sample`. Argument `source` accepts one of the following values: `"FANTOM5"`, `"FANTOM3and4"`, `"ENCODE"`, or `"ZebrafishDevelopment"`, which refer to one of the four resources listed above. The following sections explain how to utilize this function for each of the four currently supported resources.

5.2.1 FANTOM5 human and mouse samples

Lists of all human and mouse CAGE samples produced within FANTOM5 project are available in *CAGEr*. To load the information on human samples type:

```
> data(FANTOM5humanSamples)
> head(FANTOM5humanSamples)

                                                  sample
143      acantholytic_squamous_carcinoma_cell_line_HCC1806
46   acute_lymphoblastic_leukemia__B-ALL__cell_line_BALL-1
75   acute_lymphoblastic_leukemia__B-ALL__cell_line_NALM-6
24  acute_lymphoblastic_leukemia__T-ALL__cell_line_HPB-ALL
48   acute_lymphoblastic_leukemia__T-ALL__cell_line_Jurkat
250     acute_myeloid_leukemia__FAB_M0__cell_line_Kasumi-3
         type
143 cell line
46  cell line
75  cell line
24  cell line
48  cell line
250 cell line
                                             description
143      acantholytic squamous carcinoma cell line:HCC1806
46   acute lymphoblastic leukemia (B-ALL) cell line:BALL-1
```

```
75   acute lymphoblastic leukemia (B-ALL) cell line:NALM-6
24  acute lymphoblastic leukemia (T-ALL) cell line:HPB-ALL
48   acute lymphoblastic leukemia (T-ALL) cell line:Jurkat
250     acute myeloid leukemia (FAB M0) cell line:Kasumi-3
    library_id
143  CNhs11844
46   CNhs11251
75   CNhs11282
24   CNhs10746
48   CNhs11253
250  CNhs13241

143                   http://fantom.gsc.riken.jp/5/datafiles/latest/basic/human.cell_l
46     http://fantom.gsc.riken.jp/5/datafiles/latest/basic/human.cell_line.hCAGE/acute%
75     http://fantom.gsc.riken.jp/5/datafiles/latest/basic/human.cell_line.hCAGE/acute%
24    http://fantom.gsc.riken.jp/5/datafiles/latest/basic/human.cell_line.hCAGE/acute%2
48     http://fantom.gsc.riken.jp/5/datafiles/latest/basic/human.cell_line.hCAGE/acute%
250 http://fantom.gsc.riken.jp/5/datafiles/latest/basic/human.cell_line.LQhCAGE/acute%2
```

```
> nrow(FANTOM5humanSamples)
```

```
[1] 988
```

There are 988 human samples in total and for each the following information is provided:

- `sample`: a unique name/label of the sample which should be provided to `importPublicData()` function to retrieve given sample
- `type`: type of sample, which can be ”cell line”, ”primary cell” or ”tissue”
- `description`: short description of the sample as provided in FANTOM5 main publication (Consortium (2014))
- `library_id`: unique ID of the CAGE library within FANTOM5
- `data_url`: URL to corresponding CTSS file at FANTOM5 web resource from which the data is fetched

Provided information facilitates searching for samples of interest, *e.g.* we can search for astrocyte samples:

```
> astrocyteSamples <- FANTOM5humanSamples[grep("Astrocyte",
+                                   FANTOM5humanSamples[,"description"]),]
> astrocyteSamples
```

```
                              sample         type
343      Astrocyte_-_cerebellum__donor1 primary cell
537      Astrocyte_-_cerebellum__donor2 primary cell
562      Astrocyte_-_cerebellum__donor3 primary cell
286 Astrocyte_-_cerebral_cortex__donor1 primary cell
429 Astrocyte_-_cerebral_cortex__donor2 primary cell
472 Astrocyte_-_cerebral_cortex__donor3 primary cell
                           description library_id
343      Astrocyte - cerebellum, donor1  CNhs11321
537      Astrocyte - cerebellum, donor2  CNhs12081
562      Astrocyte - cerebellum, donor3  CNhs12117
286 Astrocyte - cerebral cortex, donor1  CNhs10864
429 Astrocyte - cerebral cortex, donor2  CNhs11960
472 Astrocyte - cerebral cortex, donor3  CNhs12005

343          http://fantom.gsc.riken.jp/5/datafiles/latest/basic/human.primary_cell.hCA
537          http://fantom.gsc.riken.jp/5/datafiles/latest/basic/human.primary_cell.hCA
562          http://fantom.gsc.riken.jp/5/datafiles/latest/basic/human.primary_cell.hCA
286 http://fantom.gsc.riken.jp/5/datafiles/latest/basic/human.primary_cell.hCAGE/Astroc
429 http://fantom.gsc.riken.jp/5/datafiles/latest/basic/human.primary_cell.hCAGE/Astroc
472 http://fantom.gsc.riken.jp/5/datafiles/latest/basic/human.primary_cell.hCAGE/Astroc
```

Analogous information is provided for mouse samples, which can be loaded by typing:

```
> data(FANTOM5mouseSamples)
> head(FANTOM5mouseSamples)
```

```
                                                                          sample
144 J2E_erythroblastic_leukemia_response_to_erythropoietin__00hr00min__biol_rep1
145 J2E_erythroblastic_leukemia_response_to_erythropoietin__00hr00min__biol_rep2
146 J2E_erythroblastic_leukemia_response_to_erythropoietin__00hr00min__biol_rep3
91                           Atoh1+_Inner_ear_hair_cells_-_organ_of_corti__pool1
111      CD326+_enterocyte_isolated_from_mice__treated_with_RANKL__day03__pool1
109     CD326++_enterocyte_isolated_from_mice__treated_with_RANKL__day03__pool1
            type
144    cell line
145    cell line
146    cell line
91  primary cell
111 primary cell
109 primary cell
                                                                     description
144 J2E erythroblastic leukemia response to erythropoietin, 00hr00min, biol_rep1
```

```
145 J2E erythroblastic leukemia response to erythropoietin, 00hr00min, biol_rep2
146 J2E erythroblastic leukemia response to erythropoietin, 00hr00min, biol_rep3
91                          Atoh1+ Inner ear hair cells - organ of corti, pool1
111       CD326+ enterocyte isolated from mice, treated with RANKL, day03, pool1
109      CD326++ enterocyte isolated from mice, treated with RANKL, day03, pool1
    library_id
144  CNhs12449
145  CNhs12668
146  CNhs12770
91   CNhs12533
111  CNhs13242
109  CNhs13236

144                   http://fantom.gsc.riken.jp/5/datafiles/latest/basic/mouse.timeco
145                   http://fantom.gsc.riken.jp/5/datafiles/latest/basic/mouse.timeco
146                   http://fantom.gsc.riken.jp/5/datafiles/latest/basic/mouse.timeco
91                                 http://fantom.gsc.riken.jp/5/datafiles/latest/basic
111      http://fantom.gsc.riken.jp/5/datafiles/latest/basic/mouse.primary_cell.LQhCAGE
109 http://fantom.gsc.riken.jp/5/datafiles/latest/basic/mouse.primary_cell.LQhCAGE/CD32
```

```
> nrow(FANTOM5mouseSamples)
```

```
[1] 395
```

To import TSS data for samples of interest from FANTOM5 we use the `importPublicData()` function and set the argument `source = "FANTOM5"`. The `dataset` argument can be set to either `"human"` or `"mouse"`, and the `sample` argument is provided by a vector of sample lables/names. For example, names of astrocyte samples from above are:

```
> astrocyteSamples[,"sample"]
```

```
[1] "Astrocyte_-_cerebellum__donor1"
[2] "Astrocyte_-_cerebellum__donor2"
[3] "Astrocyte_-_cerebellum__donor3"
[4] "Astrocyte_-_cerebral_cortex__donor1"
[5] "Astrocyte_-_cerebral_cortex__donor2"
[6] "Astrocyte_-_cerebral_cortex__donor3"
```

and to import first three samples type:

```
> astrocyteCAGEset <- importPublicData(source = "FANTOM5", dataset = "human",
+                                          sample = astrocyteSamples[1:3,"sample"])
> astrocyteCAGEset
```

```
S4 Object of class CAGEset

========================================
Input data information
========================================
Reference genome (organism): BSgenome.Hsapiens.UCSC.hg19
Input file type: FANTOM5
Input file names: FANTOM5__Astrocyte_-_cerebellum__donor1, FANTOM5__Astrocyte_-_cerebel
Sample labels: Astrocyte_-_cerebellum__donor1, Astrocyte_-_cerebellum__donor2, Astrocyt
========================================
CTSS information
========================================
CTSS chromosome: chr1, chr1, chr1, ...
CTSS position: 564634, 564637, 564645, ...
CTSS strand: +, +, +, ...
Tag count:
        -> Astrocyte_-_cerebellum__donor1: 0, 0, 0, ...
         -> Astrocyte_-_cerebellum__donor2: 0, 0, 0, ...
         -> Astrocyte_-_cerebellum__donor3: 1, 1, 2, ...
Normalized tpm:
========================================
Tag cluster (TC) information
========================================
CTSS clustering method:
Number of TCs per sample:
========================================
Consensus cluster information
========================================
Number of consensus clusters:
Consensus cluster chromosome:
Consensus cluster start:
Consensus cluster end:
Consensus cluster strand:
Normalized tpm:
========================================
Expression profiling
========================================
Expression clustering method:
Expression clusters for consensus clusters:
========================================
Promoter shifting
========================================
```

```
GroupX:
GroupY:
Shifting scores:
KS p-values (FDR adjusted):
```

The resulting `astrocyteCAGEset` is a `CAGEset` object that can be included in the *CAGEr* workflow described above to perform normalisation, clustering, visualisation, etc.

5.2.2 *FANTOM3and4CAGE* data package

To use TSS data from FANTOM3 and FANTOM4 projects, a data package *FANTOM3and4CAGE* has to be installed and loaded. This package is available from Bioconductor and can be installed by calling:

```
> source("http://bioconductor.org/biocLite.R")
> biocLite("FANTOM3and4CAGE")
```

For the list of available datasets with group and sample labels for specific human or mouse samples load the data package and get list of samples:

```
> library(FANTOM3and4CAGE)
> data(FANTOMhumanSamples)
> head(FANTOMhumanSamples)

                dataset           group          sample
1 FANTOMtissueCAGEhuman        cerebrum        cerebrum
2 FANTOMtissueCAGEhuman    renal_artery    renal_artery
3 FANTOMtissueCAGEhuman          ureter          ureter
4 FANTOMtissueCAGEhuman urinary_bladder urinary_bladder
5 FANTOMtissueCAGEhuman          kidney      malignancy
6 FANTOMtissueCAGEhuman          kidney          kidney

> data(FANTOMmouseSamples)
> head(FANTOMmouseSamples)

                dataset           group
1 FANTOMtissueCAGEmouse           brain
2 FANTOMtissueCAGEmouse           brain
3 FANTOMtissueCAGEmouse           brain
4 FANTOMtissueCAGEmouse           brain
5 FANTOMtissueCAGEmouse cerebral_cortex
6 FANTOMtissueCAGEmouse     hippocampus
                                           sample
```

```
1                                          brain
2                      CCL-131_Neuro-2a_control
3  CCL-131_Neuro-2a_treatment_for_6hr_with_MPP+
4 CCL-131_Neuro-2a_treatment_for_12hr_with_MPP+
5                               cerebral_cortex
6                                   hippocampus
```

In the above data frames, the columns `dataset`, `group` and `sample` provide values that should be passed to corresponding arguments in `importPublicData()` function. For example to import human kidney normal and malignancy samples call:

```
> kidneyCAGEset <- importPublicData(source = "FANTOM3and4",
+                           dataset = "FANTOMtissueCAGEhuman",
+                           group = "kidney", sample = c("kidney", "malignancy"))
> kidneyCAGEset
```

```
S4 Object of class CAGEset

=======================================
Input data information
=======================================
Reference genome (organism): BSgenome.Hsapiens.UCSC.hg18
Input file type: FANTOM3and4
Input file names: FANTOM3and4__kidney__kidney, FANTOM3and4__kidney__malignancy
Sample labels: kidney__kidney, kidney__malignancy
=======================================
CTSS information
=======================================
CTSS chromosome: chr1, chr1, chr1, ...
CTSS position: 10006, 554450, 703878, ...
CTSS strand: -, +, -, ...
Tag count:
        -> kidney__kidney: 1, 1, 1, ...
         -> kidney__malignancy: 0, 0, 0, ...
Normalized tpm:
=======================================
Tag cluster (TC) information
=======================================
CTSS clustering method:
Number of TCs per sample:
=======================================
Consensus cluster information
=======================================
```

```
Number of consensus clusters:
Consensus cluster chromosome:
Consensus cluster start:
Consensus cluster end:
Consensus cluster strand:
Normalized tpm:
========================================
Expression profiling
========================================
Expression clustering method:
Expression clusters for consensus clusters:
========================================
Promoter shifting
========================================
GroupX:
GroupY:
Shifting scores:
KS p-values (FDR adjusted):
```

When the samples belong to different groups or different datasets, it is necessary to provide the dataset and group assignment for each sample separately:

```
> mixedCAGEset <- importPublicData(source = "FANTOM3and4",
+                   dataset = c("FANTOMtissueCAGEmouse", "FANTOMtissueCAGEmouse",
+                   "FANTOMtimecourseCAGEmouse"), group = c("liver", "liver",
+                   "liver_under_constant_darkness"),
+                   sample = c("cloned_mouse", "control_mouse", "4_hr"))
> mixedCAGEset
```

```
S4 Object of class CAGEset

========================================
Input data information
========================================
Reference genome (organism): BSgenome.Mmusculus.UCSC.mm9
Input file type: FANTOM3and4
Input file names: FANTOM3and4__liver__cloned_mouse, FANTOM3and4__liver__control_mouse,
Sample labels: liver__cloned_mouse, liver__control_mouse, liver_under_constant_darkness
========================================
CTSS information
========================================
CTSS chromosome: chr1, chr1, chr1, ...
CTSS position: 3153104, 3218269, 3467930, ...
```

```
CTSS strand: +, +, +, ...
Tag count:
        -> liver__cloned_mouse: 1, 1, 1, ...
         -> liver__control_mouse: 0, 0, 0, ...
         -> liver_under_constant_darkness__4_hr: 0, 0, 0, ...
Normalized tpm:
=======================================
Tag cluster (TC) information
=======================================
CTSS clustering method:
Number of TCs per sample:
=======================================
Consensus cluster information
=======================================
Number of consensus clusters:
Consensus cluster chromosome:
Consensus cluster start:
Consensus cluster end:
Consensus cluster strand:
Normalized tpm:
=======================================
Expression profiling
=======================================
Expression clustering method:
Expression clusters for consensus clusters:
=======================================
Promoter shifting
=======================================
GroupX:
GroupY:
Shifting scores:
KS p-values (FDR adjusted):
```

For more details about datasets available in the *FANTOM3and4CAGE* data package please refer to the vignette accompanying the package.

5.2.3 *ENCODEprojectCAGE* data package

TSS data derived from ENCODE CAGE datasets has been collected into *ENCODEprojectCAGE* data package, which is available for download from the *CAGEr* web site (http://promshift.genereg.net/CAGEr/). Downloaded package can be installed from local using `install.packages()` function from within R and used with *CAGEr* as described below. List of datasets available in this data package can be obtained like this:

```
> library(ENCODEprojectCAGE)
> data(ENCODEhumanCellLinesSamples)
```

The information provided in this data frame is analogous to the one in previously discussed data package and provides values to be used with `importPublicData()` function. The command to import whole cell CAGE samples for three different cell lines would look like this:

```
> ENCODEset <- importPublicData(source = "ENCODE",
+          dataset = c("A549", "H1-hESC", "IMR90"),
+          group = c("cell", "cell", "cell"), sample = c("A549_cell_rep1",
+          "H1-hESC_cell_rep1", "IMR90_cell_rep1"))
```

For more details about datasets available in the *ENCODEprojectCAGE* data package please refer to the vignette accompanying the package.

5.2.4 *ZebrafishDevelopmentalCAGE* data package

The zebrafish TSS data for 12 developmental stages is collected in *ZebrafishDevelopmentalCAGE* data package, which is also available for download from the *CAGEr* web site (http://promshift.genereg.net/CAGEr/). It can be installed from local using `install.packages()` function. To get a list of samples within the package type:

```
> library(ZebrafishDevelopmentalCAGE)
> data(ZebrafishSamples)
```

In this package there is only one dataset called `ZebrafishCAGE` and all samples belong to the same group called `development`. To import selected samples from this dataset type:

```
> zebrafishCAGEset <- importPublicData(source = "ZebrafishDevelopment",
+                             dataset = "ZebrafishCAGE", group = "development",
+                             sample = c("zf_64cells", "zf_prim6"))
```

For more details please refer to the vignette accompanying the data package.

Importing TSS data from any of the four above explained resources results in the `CAGEset` object that can be directly included into the workflow provided by *CAGEr* to perform normalisation, clustering, promoter width analysis, visualisation, *etc.* This high-resolution TSS data can then easily be integrated with other genomic data types to perform various promoter-centred analyses, which does not rely on annotation but uses precise and matched cell/tissue type TSSs.

6 Session Info

```
> sessionInfo()

R version 3.1.0 (2014-04-10)
Platform: x86_64-apple-darwin13.1.0 (64-bit)

locale:
[1] C/C/C/C/C/en_GB.UTF-8

attached base packages:
[1] parallel  stats     graphics  grDevices utils
[6] datasets  methods   base

other attached packages:
 [1] FANTOM3and4CAGE_1.0.0
 [2] BSgenome.Drerio.UCSC.danRer7_1.3.99
 [3] CAGEr_1.9.2
 [4] BSgenome_1.32.0
 [5] Biostrings_2.32.1
 [6] XVector_0.4.0
 [7] GenomicRanges_1.16.4
 [8] GenomeInfoDb_1.0.2
 [9] IRanges_1.22.10
[10] BiocGenerics_0.10.0

loaded via a namespace (and not attached):
 [1] BBmisc_1.7               BatchJobs_1.3
 [3] BiocParallel_0.6.1       DBI_0.2-7
 [5] GenomicAlignments_1.0.5 RCurl_1.95-4.3
 [7] RSQLite_0.11.4           Rcpp_0.11.2
 [9] Rsamtools_1.16.1         VGAM_0.9-4
[11] XML_3.98-1.1             beanplot_1.1
[13] bitops_1.0-6             brew_1.0-6
[15] checkmate_1.3            codetools_0.2-8
[17] data.table_1.9.2         digest_0.6.4
[19] fail_1.2                 foreach_1.4.2
[21] iterators_1.0.7          plyr_1.8.1
[23] reshape2_1.4             rtracklayer_1.24.2
[25] sendmailR_1.1-2          som_0.3-5
[27] stats4_3.1.0             stringr_0.6.2
[29] tools_3.1.0              zlibbioc_1.10.0
```

References

Balwierz, P. J., Carninci, P., Daub, C. O., Kawai, J., Hayashizaki, Y., Van Belle, W., Beisel, C., and van Nimwegen, E. (2009). Methods for analyzing deep sequencing expression data: constructing the human and mouse promoterome with deepCAGE data. *Genome Biology*, 10(7):R79.

Carninci, P., Kvam, C., Kitamura, A., Ohsumi, T., Okazaki, Y., Itoh, M., Kamiya, M., Shibata, K., Sasaki, N., Izawa, M., Muramatsu, M., Hayashizaki, Y., and Schneider, C. (1996). High-efficiency full-length cDNA cloning by biotinylated CAP trapper. *Genomics*, 37(3):327–336.

Carninci, P., Sandelin, A., Lenhard, B., Katayama, S., Shimokawa, K., Ponjavic, J., Semple, C. A. M., Taylor, M. S., Engström, P. G., Frith, M. C., Forrest, A. R. R., Alkema, W. B., Tan, S. L., Plessy, C., Kodzius, R., Ravasi, T., Kasukawa, T., Fukuda, S., Kanamori-Katayama, M., Kitazume, Y., Kawaji, H., Kai, C., Nakamura, M., Konno, H., Nakano, K., Mottagui-Tabar, S., Arner, P., Chesi, A., Gustincich, S., Persichetti, F., Suzuki, H., Grimmond, S. M., Wells, C. A., Orlando, V., Wahlestedt, C., Liu, E. T., Harbers, M., Kawai, J., Bajic, V. B., Hume, D. A., and Hayashizaki, Y. (2006). Genome-wide analysis of mammalian promoter architecture and evolution. *Nature Genetics*, 38(6):626–635.

Consortium, T. F. (2005). The Transcriptional Landscape of the Mammalian Genome. *Science*, 309(5740):1559–1563.

Consortium, T. F. (2014). A promoter-level mammalian expression atlas. *Nature*, 507(7493):462–470.

Djebali, S., Davis, C. A., Merkel, A., Dobin, A., Lassmann, T., Mortazavi, A., Tanzer, A., Lagarde, J., Lin, W., Schlesinger, F., Xue, C., Marinov, G. K., Khatun, J., Williams, B. A., Zaleski, C., Rozowsky, J., Röder, M., Kokocinski, F., Abdelhamid, R. F., Alioto, T., Antoshechkin, I., Baer, M. T., Bar, N. S., Batut, P., Bell, K., Bell, I., Chakrabortty, S., Chen, X., Chrast, J., Curado, J., Derrien, T., Drenkow, J., Dumais, E., Dumais, J., Duttagupta, R., Falconnet, E., Fastuca, M., Fejes-Toth, K., Ferreira, P., Foissac, S., Fullwood, M. J., Gao, H., Gonzalez, D., Gordon, A., Gunawardena, H., Howald, C., Jha, S., Johnson, R., Kapranov, P., King, B., Kingswood, C., Luo, O. J., Park, E., Persaud, K., Preall, J. B., Ribeca, P., Risk, B., Robyr, D., Sammeth, M., Schaffer, L., See, L.-H., Shahab, A., Skancke, J., Suzuki, A. M., Takahashi, H., Tilgner, H., Trout, D., Walters, N., Wang, H., Wrobel, J., Yu, Y., Ruan, X., Hayashizaki, Y., Harrow, J., Gerstein, M., Hubbard, T., Reymond, A., Antonarakis, S. E., Hannon, G., Giddings, M. C., Ruan, Y., Wold, B., Carninci, P., Guigo, R., and Gingeras, T. R. (2012). Landscape of transcription in human cells. *Nature*, 488(7414):101–108.

Faulkner, G. J., Kimura, Y., Daub, C. O., Wani, S., Plessy, C., Irvine, K. M., Schroder, K., Cloonan, N., Steptoe, A. L., Lassmann, T., Waki, K., Hornig, N., Arakawa, T.,

Takahashi, H., Kawai, J., Forrest, A. R. R., Suzuki, H., Hayashizaki, Y., Hume, D. A., Orlando, V., Grimmond, S. M., and Carninci, P. (2009). The regulated retrotransposon transcriptome of mammalian cells. *Nature Genetics*, 41(5):563–571.

Frith, M. C., Valen, E., Krogh, A., Hayashizaki, Y., Carninci, P., and Sandelin, A. (2007). A code for transcription initiation in mammalian genomes. *Genome Research*, 18(1):1–12.

Haberle, V., Li, N., Hadzhiev, Y., Plessy, C., Previti, C., Nepal, C., Gehrig, J., Dong, X., Akalin, A., Suzuki, A. M., van IJcken, W. F. J., Armant, O., Ferg, M., Strähle, U., Carninci, P., Müller, F., and Lenhard, B. (2014). Two independent transcription initiation codes overlap on vertebrate core promoters. *Nature*, 507(7492):381–385.

Kodzius, R., Kojima, M., Nishiyori, H., Nakamura, M., Fukuda, S., Tagami, M., Sasaki, D., Imamura, K., Kai, C., Harbers, M., Hayashizaki, Y., and Carninci, P. (2006). CAGE: cap analysis of gene expression. *Nature Methods*, 3(3):211–222.

Nepal, C., Hadzhiev, Y., Previti, C., Haberle, V., Li, N., Takahashi, H., Suzuki, A. M. S., Sheng, Y., Abdelhamid, R. F., Anand, S., Gehrig, J., Akalin, A., Kockx, C. E., van der Sloot, A. A., van IJcken, W. F., Armant, O., Rastegar, S., Watson, C., Strähle, U., Stupka, E., Carninci, P., Lenhard, B., and Müller, F. (2013). Dynamic regulation of coding and non-coding transcription initiation landscape at single nucleotide resolution during vertebrate embryogenesis. *Genome Research*, 23(11):1938–1950.

Suzuki, H., Forrest, A. R. R., van Nimwegen, E., Daub, C. O., Balwierz, P. J., Irvine, K. M., Lassmann, T., Ravasi, T., Hasegawa, Y., de Hoon, M. J. L., Katayama, S., Schroder, K., Carninci, P., Tomaru, Y., Kanamori-Katayama, M., Kubosaki, A., Akalin, A., Ando, Y., Arner, E., Asada, M., Asahara, H., Bailey, T., Bajic, V. B., Bauer, D., Beckhouse, A. G., Bertin, N., Björkegren, J., Brombacher, F., Bulger, E., Chalk, A. M., Chiba, J., Cloonan, N., Dawe, A., Dostie, J., Engström, P. G., Essack, M., Faulkner, G. J., Fink, J. L., Fredman, D., Fujimori, K., Furuno, M., Gojobori, T., Gough, J., Grimmond, S. M., Gustafsson, M., Hashimoto, M., Hashimoto, T., Hatakeyama, M., Heinzel, S., Hide, W., Hofmann, O., Hörnquist, M., Huminiecki, L., Ikeo, K., Imamoto, N., Inoue, S., Inoue, Y., Ishihara, R., Iwayanagi, T., Jacobsen, A., Kaur, M., Kawaji, H., Kerr, M. C., Kimura, R., Kimura, S., Kimura, Y., Kitano, H., Koga, H., Kojima, T., Kondo, S., Konno, T., Krogh, A., Kruger, A., Kumar, A., Lenhard, B., Lennartsson, A., Lindow, M., Lizio, M., MacPherson, C., Maeda, N., Maher, C. A., Maqungo, M., Mar, J., Matigian, N. A., Matsuda, H., Mattick, J. S., Meier, S., Miyamoto, S., Miyamoto-Sato, E., Nakabayashi, K., Nakachi, Y., Nakano, M., Nygaard, S., Okayama, T., Okazaki, Y., Okuda-Yabukami, H., Orlando, V., Otomo, J., Pachkov, M., Petrovsky, N., Plessy, C., Quackenbush, J., Radovanovic, A., Rehli, M., Saito, R., Sandelin, A., Schmeier, S., Schönbach, C., Schwartz, A. S., Semple, C. A., Sera, M., Severin, J., Shirahige, K., Simons, C., St Laurent, G., Suzuki, M., Suzuki, T., Sweet, M. J., Taft, R. J., Takeda, S., Takenaka, Y., Tan, K., Taylor,

M. S., Teasdale, R. D., Tegnér, J., Teichmann, S., Valen, E., Wahlestedt, C., Waki, K., Waterhouse, A., Wells, C. A., Winther, O., Wu, L., Yamaguchi, K., Yanagawa, H., Yasuda, J., Zavolan, M., Hume, D. A., Arakawa, T., Fukuda, S., Imamura, K., Kai, C., Kaiho, A., Kawashima, T., Kawazu, C., Kitazume, Y., Kojima, M., Miura, H., Murakami, K., Murata, M., Ninomiya, N., Nishiyori, H., Noma, S., Ogawa, C., Sano, T., Simon, C., Tagami, M., Takahashi, Y., Kawai, J., and Hayashizaki, Y. (2009). The transcriptional network that controls growth arrest and differentiation in a human myeloid leukemia cell line. *Nature Genetics*, 41(5):553–562.

Takahashi, H., Lassmann, T., Murata, M., and Carninci, P. (2012). 5' end-centered expression profiling using cap-analysis gene expression and next-generation sequencing. *Nature Protocols*, 7(3):542–561.

R code for processing mouse testis development time-course data with *CAGEr* and performing analyses presented in the paper

```
#-------------------------------------------------------------------------------------#
# Installing CAGEr package
#-------------------------------------------------------------------------------------#

# install latest version of CAGEr and all dependencies from Bioconductor
source("http://bioconductor.org/biocLite.R")
biocLite("CAGEr")

# load CAGEr package into current R session
library(CAGEr)


#-------------------------------------------------------------------------------------#
# Reading in CAGE TSS data for 8 mouse testis samples
#-------------------------------------------------------------------------------------#

# download input TSS file from http://promshift.genereg.net/CAGEr/InputData/
wget
http://promshift.genereg.net/CAGEr/InputData/CTSS_mouse_testis_timecourse_8_samples.txt

# specify path to input file and names of samples – tab separated file with
# first 3 columns specifying genomic coordinates of TSSs:
# chromosome, 1-based coordinate, strand
# followed by 8 columns with tag counts in 8 mouse testis samples
input.file <- "CTSS_mouse_testis_timecourse_8_samples.txt"
samples <- c(paste("E", c(13,15,17), sep = ""), paste("N", c("00",10,20,30),
             sep = ""), "adult")

# initialize an empty CAGEset object and read in the data
myCAGEset <- new("CAGEset", genomeName = "BSgenome.Mmusculus.UCSC.mm9",
                 inputFiles = input.file, inputFilesType = "CTSStable",
                 sampleLabels = samples)
getCTSS(myCAGEset)

# setting colors that will be used in plots and tracks to represent
# the 8 different samples
setColors(myCAGEset, colors = c("midnightblue", "royalblue", "cyan4", "yellow4",
                               "goldenrod", "darkorange2", "red3", "firebrick4"))


#-------------------------------------------------------------------------------------#
# Supplementary Figure S1:  Visualisation of TSSs and correlation between samples
#-------------------------------------------------------------------------------------#

# export single base-pair resolution and strand-specific TSS data into
# bedGraph file for visualisation in the genome browser (Supplementary Figure S1b)
exportCTSStoBedGraph(myCAGEset, values = "raw", oneFile = TRUE)

# plot pair-wise scatter plots and calculate correlation (Supplementary Figure S1c)
corr.matrix <- plotCorrelation(myCAGEset, what = "CTSS", values = "raw")


#-------------------------------------------------------------------------------------#
# Figure 2:  Reverse cumulatives and normalisation to a referent power-law distribution
#-------------------------------------------------------------------------------------#

# reverse cumulatives for raw tag counts
plotReverseCumulatives(myCAGEset, values = "raw", fitInRange = c(10,1000), onePlot=T)

# normalisation
normalizeTagCount(myCAGEset, method = "powerLaw", fitInRange = c(10,1000),
                  alpha = 1.27, T = 10^6)
```

```
# reverse cumulatives for normalised tag counts
plotReverseCumulatives(myCAGEset, values = "normalized", onePlot=T)


#------------------------------------------------------------------------------------#
# Supplementary Figure S2:  clustering TSSs into TCs (TSS clusters)
#------------------------------------------------------------------------------------#

# simple distance-based clustering
# advisable to run in parallel (useMulticore = TRUE)
clusterCTSS(object = myCAGEset, method = "distclu", maxDist = 20, removeSingletons =
            TRUE, keepSingletonsAbove = 5, useMulticore = TRUE, nrCores = 8)

# export tag clusters (TCs) per sample into BED file for visualisation
# in the genome browser
exportToBed(object = myCAGEset, what = "tagClusters", oneFile = TRUE)


# paraclu - parametric clustering (Frith et al. Genome Research 2008)
# advisable to run in parallel (useMulticore = TRUE)
paracluCAGEset <- myCAGEset

# overlapping clusters
clusterCTSS(object = paracluCAGEset, method = "paraclu", removeSingletons = TRUE,
            keepSingletonsAbove = 5, minStability = 1, maxLength = 500,
            reduceToNonoverlapping = FALSE, useMulticore = TRUE, nrCores = 8)

# non-overlapping clusters
clusterCTSS(object = paracluCAGEset, method = "paraclu", removeSingletons = TRUE,
            keepSingletonsAbove = 5, minStability = 1, maxLength = 500,
            reduceToNonoverlapping = TRUE, useMulticore = TRUE, nrCores = 8)

# export paraclu tag clusters (TCs) per sample into BED file for visualisation
# in the genome browser
exportToBed(object = paracluCAGEset, what = "tagClusters", oneFile = TRUE)


#------------------------------------------------------------------------------------#
# Figure 3 and Supplementary Figure S3:  Promoter (TC) width
#------------------------------------------------------------------------------------#

# calculate cumulative distribution of CAGE signal along tag clusters
# advisable to run in parallel (useMulticore = TRUE)
cumulativeCTSSdistribution(object = myCAGEset, clusters = "tagClusters",
                           useMulticore = TRUE, nrCores = 8)

# determine positions of 10th and 90th percentile of the signal in each cluster
# advisable to run in parallel (useMulticore = TRUE)
quantilePositions(object = myCAGEset, clusters = "tagClusters", qLow = 0.1, qUp = 0.9,
                  useMulticore = TRUE, nrCores = 8)

# plotting distribution of interquantile width per sample (Figure S3b)
plotInterquantileWidth(object = myCAGEset, clusters = "tagClusters", tpmThreshold = 5,
                       qLow = 0.1, qUp = 0.9)

# export individual TCs interquantile width into BED file for visualisation in the
# genome browser (Supplementary Figure S3a)
exportToBed(object = myCAGEset, what = "tagClusters", qLow = 0.1, qUp = 0.9,
            oneFile = TRUE)

# getting list of sharp and broad TCs in mouse testis used for analyses
# presented in Figure 3 and Figure 6
tc <- tagClusters(myCAGEset, sample = "N30", returnInterquantileWidth = TRUE,
                  qLow = 0.1, qUp = 0.9)
tc.sharp <- subset(tc, interquantile_width < 10.5 & tpm >= 5)
tc.broad <- subset(tc, interquantile_width > 10.5 & tpm >= 5)
```

```
#--------------------------------------------------------------------------------------#
# Creating consensus clusters
#--------------------------------------------------------------------------------------#

# aggregate TCs across 8 samples into one set of consensus clusters
aggregateTagClusters(object = myCAGEset, tpmThreshold = 5, excludeSignalBelowThreshold
                     = FALSE, qLow = 0.1, qUp = 0.9, maxDist = 100)


#--------------------------------------------------------------------------------------#
# Figure 4 and Supplementary Figure S4:  Expression profiles of promoters and
#                                        individual TSSs
#--------------------------------------------------------------------------------------#

# expression clustering of individual TSSs using self-organising maps algorithm
getExpressionProfiles(object = myCAGEset, what = "CTSS", tpmThreshold = 5,
                      nrPassThreshold = 1, method = "som", xDim = 2, yDim = 4)

# plotting SOM map with beanplots (Supplementary Figure S4b)
plotExpressionProfiles(object = myCAGEset, what = "CTSS")

# export CTSSs colored by expression class into BED file for visualisation in the
# genome browser (Supplementary Figure S4c)
exportToBed(object = myCAGEset, what = "CTSS", colorByExpressionProfile = TRUE)


# expression clustering of entire promoters (consensus clusters) using self-organising
# maps algorithm
getExpressionProfiles(object = myCAGEset, what = "consensusClusters", tpmThreshold =
                      5, nrPassThreshold = 1, method = "som", xDim = 2, yDim = 4)

# plotting SOM map with beanplots (Figure 4a)
plotExpressionProfiles(object = myCAGEset, what = "consensusClusters")

# export promoters colored by expression class into BED file for visualisation in the
# genome browser (Figure 4b, Supplementary Figure S4a)
exportToBed(object = myCAGEset, what = "consensusClusters",
            colorByExpressionProfile = TRUE)


#--------------------------------------------------------------------------------------#
# Figure 5 and Supplementary Figure S5:  Differential TSS usage and shifting promoters
#--------------------------------------------------------------------------------------#

# calculate cumulative distribution of CAGE signal along consensus clusters
# advisable to run in parallel (useMulticore = TRUE)
cumulativeCTSSdistribution(object = myCAGEset, clusters = "consensusClusters",
                           useMulticore = TRUE, nrCores = 8)

# calculating shifting score and assessing significance of differential TSS usage
# between embryonic day 13 and adult testis
# advisable to run in parallel (useMulticore = TRUE)
scoreShift(object = myCAGEset, groupX = "adult", groupY = "E13",
           testKS = TRUE, useMulticore = TRUE, nrCores = 8)

# list of all promoters with significant differential TSS usage (Supplementary Table 2)
diff.TSS.promoters <- getShiftingPromoters(object = myCAGEset, tpmThreshold = 5,
                                           fdrThreshold = 0.01)

# list of "shifting" promoters with min 40% spatial separation in TSS usage
# (example in Figure 5c)
shifting.promoters <- subset(diff.TSS.promoters, shifting.score >= 0.4)
```

Examples of R code demonstrating retrieval of TSS data for selected samples from available resources into *CAGEr* workflow

```
#-------------------------------------------------------------------------------------#
# Retrieving selected samples from FANTOM5 collection
#-------------------------------------------------------------------------------------#

# load CAGEr package into current R session
library(CAGEr)

# lists of all human and mouse CAGE samples produced within FANTOM5 project are
# available in CAGEr
# To load them type:
data(FANTOM5humanSamples)
data(FANTOM5mouseSamples)

# sample information is given in 5 columns, the first column provides sample name that
# should be used to retrieve given sample
head(FANTOM5humanSamples)

# search for “Astrocyte” samples in the list
astrocyteSamples <- FANTOM5humanSamples[grep("Astrocyte",
                    FANTOM5humanSamples[,"description"]),]

# import Astrocyte samples into CAGEset object to include it into CAGEr workflow
astrocyteCAGEset <- importPublicData(source = "FANTOM5", dataset = "human",
                                     sample = astrocyteSamples[,"sample"])

# the resulting astrocyteCAGEset is a CAGEset object that can be included in the
# CAGEr workflow to perform normalisation, clustering, visualisation,
# etc. as described above and in the package vignette


#-------------------------------------------------------------------------------------#
# Retrieving selected samples from FANTOM3and4CAGE R data package
#-------------------------------------------------------------------------------------#

# download and install FANTOM3and4CAGE data package from Bioconductor
source("http://bioconductor.org/biocLite.R")
biocLite("FANTOM3and4CAGE")

# load packages into current R session
library(CAGEr)
library(FANTOM3and4CAGE)

# get list of available datasets in this package
data(FANTOMhumanSamples)
head(FANTOMhumanSamples)

# import TSS data for human kidney into CAGEset object for processing with CAGEr
kidneyCAGEset <- importPublicData(source = "FANTOM3and4", dataset =
                                  "FANTOMtissueCAGEhuman", group = "kidney",
                                  sample = c("kidney", "malignancy"))
kidneyCAGEset
```

```
#------------------------------------------------------------------------------------#
# Retrieving selected samples from ENCODEprojectCAGE R data package
#------------------------------------------------------------------------------------#

# this package is available for download from
# http://promshift.genereg.net/CAGEr/PackageSource/ENCODEprojectCAGE_1.0.0.tar.gz
wget http://promshift.genereg.net/CAGEr/PackageSource/ENCODEprojectCAGE_1.0.0.tar.gz

# install downloaded package in R from local
install.packages(pkgs = "ENCODEprojectCAGE_1.0.0.tar.gz", repos = NULL, type = "source")


# load packages into current R session
library(CAGEr)
library(ENCODEprojectCAGE)

# get list of available datasets in this package
data(ENCODEhumanCellLinesSamples)
head(ENCODEhumanCellLinesSamples)

# import TSS data for three cell lines into CAGEset object for processing with CAGEr
ENCODEset <- importPublicData(source = "ENCODE", dataset = c("A549", "H1-hESC",
                              "IMR90"), group = c("cell", "cell", "cell"),
                              sample = c("A549_cell_rep1", "H1-hESC_cell_rep1",
                              "IMR90_cell_rep1"))


#------------------------------------------------------------------------------------#
# Retrieving selected samples from ZebrafishDevelopmentalCAGE R data package
#------------------------------------------------------------------------------------#

# this package is available for download from
# http://promshift.genereg.net/CAGEr/PackageSource/
wget
http://promshift.genereg.net/CAGEr/PackageSource/ZebrafishDevelopmentalCAGE_0.99.0.tar.gz

# install downloaded package in R from local
install.packages(pkgs = "ZebrafishDevelopmentalCAGE_0.99.0.tar.gz", repos = NULL,
                 type = "source")

# load packages into current R session
library(CAGEr)
library(ZebrafishDevelopmentalCAGE)

# get list of available datasets in this package
data(ZebrafishSamples)
ZebrafishSamples

# import TSS data for two zebrafish developmental stages into CAGEset object for
# processing with CAGEr
zebrafishCAGEset <- importPublicData(source = "ZebrafishDevelopment", dataset =
                                     "ZebrafishCAGE", group = "development",
                                     sample = c("zf_64cells", "zf_prim6"))
```
